# Dog-assisted interventions for adults diagnosed with schizophrenia and related disorders: a systematic review

**DOI:** 10.3389/fpsyt.2023.1192075

**Published:** 2023-06-23

**Authors:** Malene Kalsnes Tyssedal, Erik Johnsen, Aurora Brønstad, Silje Skrede

**Affiliations:** ^1^Department of Clinical Medicine, Faculty of Medicine, University of Bergen, Bergen, Norway; ^2^Division of Psychiatry, Haukeland University Hospital, Bergen, Norway; ^3^NORMENT Centre of Excellence, Haukeland University Hospital, Bergen, Norway; ^4^Department of Clinical Science, Faculty of Medicine, University of Bergen, Bergen, Norway; ^5^Section of Clinical Pharmacology, Department of Medical Biochemistry and Pharmacology, Haukeland University Hospital, Bergen, Norway

**Keywords:** animal-assisted interventions, therapy dog, PANSS, psychosocial outcomes, psychosis, severe mental illness

## Abstract

**Background:**

Many individuals diagnosed with schizophrenia and related disorders experience insufficient symptom relief from currently available treatment options. Researching additional venues should be prioritized. This systematic review, designed in accordance with PRISMA, examined the effect of targeted and structured dog-assisted interventions as a supplementary treatment.

**Methods:**

Randomized as well as non-randomized studies were included. Systematic searches were conducted in APA PsycInfo, AMED, CENTRAL, Cinahl, Embase, Medline, Web of Science, and in several sources covering “gray” (unpublished) literature. In addition, forward and backward citation searches were performed. A narrative synthesis was conducted. Quality of evidence and risk of bias were assessed in accordance with GRADE and RoB2/ROBINS-I criteria.

**Results:**

12 publications from 11 different studies met eligibility criteria. Overall, studies showed diverging results. General psychopathology, positive and negative symptoms of psychosis, anxiety, stress, self-esteem, self-determination, lower body strength, social function, and quality of life were among the outcome measures with significant improvement. Most documentation for significant improvement was found for positive symptoms. One study indicated significant deterioration of non-personal social behavior. The risk of bias was high or serious for most of the outcome measures. Three outcome measures were associated with some concerns regarding risk of bias, and three with low risk of bias. Quality of evidence was graded low or very low for all outcome measures.

**Conclusions:**

The included studies indicate potential effects of dog-assisted interventions for adults diagnosed with schizophrenia and related disorders, mostly beneficial. Nevertheless, low number of participants, heterogeneity, and risk of bias complicate the interpretation of results. Carefully designed randomized controlled trials are needed to determine causality between interventions and treatment effects.

## 1. Introduction

Schizophrenia and related psychotic disorders are characterized by positive symptoms, negative symptoms, and cognitive difficulties. Hallucinations, delusions, and disorganized speech are examples of positive symptoms, while amotivation, anhedonia, and affective flattening are examples of negative symptoms. Genetic predisposition, substance use, trauma, and acute stress are among the risk factors for development of severe psychotic disorders. In addition, neurobiological factors, such as dopamine dysfunction, are associated with presence of positive symptoms and negative symptoms, as well as cognitive difficulties (1, 2). Overall lifetime prevalence for schizophrenia and related disorders is stated as 7.49 per 1,000 (3). The prognosis varies among individuals and extends between recovery and a chronic, lifelong course (4). Life expectancy is reduced by several years, with somatic comorbidity as one of the major causes (5). Overall, severe psychotic disorders are associated with a high burden of disease (6).

Treatment recommendations consist of a combination of pharmacological and non-pharmacological interventions (7). Current antipsychotic medications are shown to be more effective for positive symptoms than for negative and cognitive symptoms (1), and the latter two symptom groups are important determinants of disability (8). Numerous non-pharmacological interventions are considered in the guidelines by the *Norwegian Directorate of Health* (7), in accordance with international standards. Psychoeducation, family interventions, cognitive therapy, physical activity, and music therapy are among the included options. However, a substantial group of individuals diagnosed with schizophrenia and related disorders do not experience sufficient symptom relief (9). The heterogenous pathophysiology and phenotypes of severe psychotic disorders underpin the need for varied treatment options (10). Direct interpersonal engagement can be too demanding in some individuals. Interaction with therapeutic animals might theoretically be a less stressful alternative.

Animals have been included in the treatment for several disorders through centuries (11). Currently, there has been a development where anecdotal evidence to a larger extent is replaced by scientific research (12). The *International Association of Human-Animal Interaction Organizations* (IAHAIO) (13) has published specific guidelines for animal-assisted interventions (AAI). These guidelines are stating that AAI must be targeted and structured, with therapeutic benefits as purpose. Animal-assisted therapy (AAT) and animal-assisted activity (AAA) are two examples of AAI relevant to health care. While AAT must be planned, measurable, and documented, AAA signifies informal interaction. The guidelines are further stating that AAT is targeted toward physical, cognitive, behavioral, and/or socio-emotional functioning, while AAA is targeted toward motivation, education, and/or recreation. Knowledge related to health and behavior of included animals is required for providers of both AAT and AAA. Professional expertise, for example within health care, is in addition required for providers of AAT.

Studies have suggested treatment effects related to AAI for a range of health conditions and diseases (14). Biophilia, stress buffering, and distraction are elements in some theories and hypotheses that may explain potential effects (15). The biophilia hypothesis describes the affinity of humans to other living species (16). Effects related to the biophilia hypothesis may involve feelings of safety and facilitation of interpersonal interactions where animals may serve as social catalysators (17). In addition, decreased levels of cortisol and increased levels of oxytocin, β-endorphin, prolactin, phenyl acetic acid, and dopamine have been detected after interaction with dogs (18). These changes may be associated with physiological and psychosocial benefits, such as stress relief and improvement of social bonding and learning (18–21). Summarized, AAI are aimed at a wide range of symptoms and features, including those presented in severe psychotic disorders. Increased motivation for therapeutic activities due to interaction with animals has been described, for example in a study including individuals with acquired brain injury (22). Treatment effects of AAI will be highly relevant to investigate further for individuals with severe psychotic disorders. This is particularly justified by the fact that lack of motivation, which affects adherence to treatment, is a core feature among the negative symptoms (23).

A systematic review (SR) from 2018 on equine-assisted interventions indicated potential effects for individuals diagnosed with schizophrenia and related disorders. Significant improvement was shown for several outcome measures, such as negative symptoms, social functioning, pharmacological compliance, and risk of violence. The authors stated that further research is needed (24). A SR from 2019, including randomized controlled trials (RCTs) on AAI with several animal species, found inconclusive results regarding treatment effects for individuals diagnosed with schizophrenia and related disorders. However, potential benefits were found for some outcome measures, such as positive symptoms, negative symptoms, emotional symptoms, and self-view (25).

As different animal species have different properties, we sought to investigate effects of dog-assisted interventions (DAI) specifically to increase directness and complement previous SRs. An investigation of therapeutic effects of DAI is also relevant due to findings in a survey among individuals diagnosed with schizophrenia, indicating that the dog was a preferred animal (26). A meta-analysis found that dogs were the most commonly involved animal in AAT (27). Beneficial therapeutic effects may be related to the cognitive and emotional capacities in dogs, in addition to an evolutionary connection with humans (28). Feasibility is also an important issue as dogs can thrive in same environments as humans. We sought to evaluate effects of targeted and structured interventions with therapeutic benefits as purpose. Therefore, both AAT and AAA were included.

Due to an existing knowledge gap, in addition to an extension of the field by four articles published during 2021–2023 (29–32), we found it relevant to perform a modified and updated SR on the topic. Summarized, modifications consisted of broader inclusion regarding study designs, and a narrower approach regarding the objective. The aim of the SR was to investigate effect of DAI for adults diagnosed with shizophrenia and related disorders. To our knowledge, this isolated topic has not been specifically covered by previous SRs.

## 2. Methods

The SR was designed in accordance with PRISMA (Preferred Reporting Items for Systematic Reviews and Meta-Analyses) guidelines (33). In addition, a document with examples from the guidelines was used (34). Two handbooks, by Cochrane (35) and by the Norwegian Institute of Public Health (NIPH) (36), were also used as references.

### 2.1. Eligibility criteria

Eligibility criteria are presented in Table 1.

**Table 1:** Eligibility criteria.

	**Inclusion criteria**	**Exclusion criteria**
Population	Participants aged 18 years or older diagnosed with schizophrenia or related disorders (37 )^a^ Ongoing treatment in a psychiatric ward, outpatient clinic or residential institution	Lack of distinguishing between measurements from participants with other diagnoses than schizophrenia and related disorders
Intervention	Dog-assisted interventions with aim of therapeutic benefits	Lack of distinguishing between measurements from interventions with different animal species
Outcome	Outcomes measured with validated instruments on at least two time points throughout the study	N/A
Study design	Quantitative studies of all designs	N/A
Report properties	Both published articles and gray literature No restrictions regarding year of publication	Articles written in other languages than English or Scandinavian
Risk of bias	N/A	Critical risk of bias

N/A, Not applicable.

^a^Schizophrenia and related disorders: Schizophrenia, schizotypal and delusional disorders (F20-F29) in International Classification of Diseases, ICD-10, and corresponding diagnoses in Diagnostic Statistical Manual, DSM-IV and DSM-V, assessed using a tool from New Zealand Health Information Service. Nevertheless, studies were not excluded due to missing diagnosis codes.

### 2.2. Search strategies, information sources and study selection

A detailed description of the search strategies can be found in Supplementary Tables 2–13. Briefly, the search strategy was developed in accordance with chapter 4 in Cochrane's method book (38) and chapter 4 in the method book by NIPH (36). Furthermore, two SRs (24, 25) on related topics, in addition to IAHAIOs definition of animal-assisted interventions (13), were used as references. Relevant articles detected through initial, non-systematic searches in Google Scholar and PubMed were reviewed for additional search terms and used for validation of the search strategy (29, 39–45).

The main searches were conducted 21.05.22 in APA PsycInfo (Ovid), AMED (Ovid), CENTRAL (Cochrane), Cinahl (Ebsco), Embase (Ovid), Medline (Ovid) and Web of Science. Automatic alerts regarding new publications until submission were set up. Duplicates from the main search were initially removed by automatic duplicate detection in EndNote version 20. Remaining duplicates were removed manually. Title and abstracts of all the remaining articles were screened by two reviewers working independently (by AB and EJ from A to K, and by MT and SS from L to AA, sorted by authors last name). Articles were initially excluded if the title or abstract did not include DAI or AAI not further specified, and schizophrenia, other psychotic disorders or mental disorders not specified.

The assessments of which articles to read in full text version and which to include in the SR, were also made independently by two reviewers for each study. The supplementary searches were conducted in the period from 30.04.22 to 28.05.23. For supplementary sources, please refer to the detailed description found in the Supplementary material. These searches consisted of both forward and backward reference searching, in addition to searches in databases, registers, and in websites of organizations. Backward citation searches in relevant reviews were conducted by EJ from A to K, and by MT from L to AA, sorted by authors last name. Beyond this, the supplementary searches were conducted by one reviewer (MT).

### 2.3. Data collection and synthesis of results

Study properties were collected in accordance with the PICO (population, intervention, comparison, outcome) model (46). Report properties were also collected, in addition to information regarding study design. Measurements regarding overall change, final values and/or follow-up for all outcomes related to effects were sought for extraction. Some of the elements were not documented in all articles. The data elements presented in Tables 2–**4** were collected by one reviewer (MT) and controlled by one reviewer (EJ). Supplementary Table 15 provides an overview over data elements sought for extraction.

**Table 2:** Study characteristics.

	**Population**	**Intervention**	**Comparison**	**Outcome measure**	**Study design**
Barker and Dawson, 1998 (47)	Schizophrenia, schizoaffective disorder and other psychotic disorders, acute Inpatients Sex^a^: F 174, M 139 Age^b^: Mean 37 years, SD 12	*n =* 34 participants (26 included in analyses) Dog-assisted *therapy* 30 min x1	*n =* 45 participants (39 included in analyses) Therapeutic recreation group session (music and art activities, education about leisure time and resources) x1 (duration not specified)	STAI (anxiety)	Crossover design
Calvo et al., 2016 (39)	Schizophrenia, chronic (DSM-IV-TR) Inpatients Sex: F 7, M 17 Age: Mean 47.8 years, SD 6.7 Age at diagnosis: Mean 20.5 years, SD 5.0	*n =* 16 participants (14 included in analyses) Dog-assisted *therapy* in addition to psychosocial rehabilitation program 6 months, 60 min x2 per week	*n =* 8 participants (8 included in analyses) One activity from the functional program (art therapy, group sports, dynamic psycho-stimulation or gymnastics) in addition to other programs of the psychosocial rehabilitation program 6 months, 60 min x2 per week	Primary outcomes: PANSS (positive, negative, general symptoms) EQ-5D (quality of life) Secondary outcomes: Adherence (patient experience) Salivary cortisol, alpha-amylase (stress relief)	RCT (non-blinded)
Chen et al., 2021 (29)	Schizophrenia, chronic (DSM-5) Inpatients and day care patients Sex: F 22, M 18 Age ≥ 40 years (mean 54.7)	*n =* 20 participants (20 included in analyses) Dog-assisted *therapy* in addition to usual treatment programs 12 weeks, 60–65 min x1 per week	*n =* 20 participants (20 included in analyses) Addition of nursing intervention and occupational therapy from the usual treatment program 12 weeks, 60 min x1 per week	Primary outcomes: PANSS (negative and general symptoms) DASS-21 (depression, anxiety, stress) Secondary outcomes: PANSS (positive symptoms) CHI (well-being)	RCT (non-blinded)
Chen et al., 2022 (30)	Same population as Chen et al., 2021 (29)	Same intervention as Chen et al., 2021 (29)	Same comparison as Chen et al., 2021 (29)	MoCa (global cognitive function) CST (lower body strength) TUG (agility) 5MWT (mobility) ACIS (communication and interaction skills)	Same design as Chen et al., 2021 (29)
Chu et al., 2009 (45)	Schizophrenia Inpatients Sex:? (authors state no significant difference between groups) Age < 60 years Duration of illness >10 years	*n =* 15 participants (12 included in analyses) Dog-assisted *activity* 8 weeks, 50 min x 1 per week	*n =* 15 participants (15 included in analyses) Treatment as usual	Questionnaire: Self-esteem Self-determination Extent of social support Adverse psychiatric symptoms (positive, negative and emotional)	RCT (assessment blinded)
Kovacs et al., 2004 (40)	Schizophrenia, chronic (DSM-IV) Inpatients Sex: F 4, M 3 Age 29–58 years (mean 43.6) Duration of illness >10 years	*n =* 7 participants (7 included in analyses) Dog-assisted *therapy* 9 months, 50 min x 1 per week	N/A	ILSS (living skills)	Pilot study (pre-post)
Kovacs et al., 2006 (41)	Schizophrenia, chronic (DSM-IV) Day-care Sex: F 3, M 2 Age: 32–71 years	*n =* 5 participants (3 included in the analyses) Dog-assisted *therapy* 6 months, 50 min x1 per week	N/A	BGRS (non-verbal communication)	Exploratory study (pre-post)
Lang et al., 2010 (42)	Schizophrenia, acute (DSM-IV) Inpatients? Sex: F 7, M 7 Age: Mean 37.3 years, SD 13.8 Duration of illness: Mean 6 years, SD 9	*n =* 14 participants (14 included in analyses) Dog-assisted *interview* 30 min x 1	*n =* 14 participants (14 included in analyses) Interview without dog 30 min x1	STAI (anxiety)	Crossover design
Monfort et al., 2022 (31)	Schizophrenia-spectrum disorders and substance-use disorders (dual pathology) Residential treatment Age: Mean 40.3 years, SD 6.1 Sex: F: 13.9%, M: 86.1%	*n =* 18 participants (13 included in analyses)^c^ Dog-assisted *therapy* in addition to standard treatment Maximum of 12 weeks (10 sessions), 45 min per week	*n =* 13 participants (10 included in analyses)^d^ Standard treatment (antipsychotics, psychotherapy, psychoeducation, cognitive therapy)	LSP-20 (life skills) PANSS (positive, negative, and general symptoms) (Lack of baseline recordings^e^)	Quasi-experimental prospective study
Nathans Barel et al., 2005 (43)	Schizophrenia, chronic (DSM-IV) Inpatients Sex: F 8, M 12 Age: Mean 39.9 years, SD 11.67 Duration of illness: Mean 18.1 years, SD 11.2	*n =* 10 participants (lost to follow-up not specified) Dog-assisted *therapy* in addition to psychosocial treatment 10 weeks x 60 min per week	*n =* 10 participants (lost to follow-up not specified) Learning about caring for animals, going for walks and participating in discussions in addition to psychosocial treatment 10 weeks x 60 min per week	Primary outcome SHAPS (anhedonia) Secondary outcomes SANS (negative symptoms) PANSS (total) PANSS (positive symptoms) SQLS (quality of life in relation to treatment) QLESQ (quality of life)	Controlled pilot study
Shih et al., 2023 (32)	Schizophrenia, chronic (DSM-5)Inpatients Sex: F 45, M 45 Age: Mean 50.2, SD 9.6 Age of morbidity: Mean 30.6, SD 11.0	*n =* 45 participants (45 included in analyses) Dog-assisted *therapy* 12 weeks, 60 min x1 per week	*n =* 45 participants (45 included in analyses) Discussion groups, including films about animals 12 weeks, 60 min x1 per week	MHSFS (Social competence and abilities in daily life) SAFS (day-to-day living abilities, social functioning, occupational abilities) WHOQOL-BREF (quality of life)	Longitudinal, single-blind experimental study
Villalta-Gil et al., 2009 (44)	Schizophrenia, (DSM-IV) Inpatients, long term Sex: F 3, M:18 Age in intervention group: Mean 49.1 years, SD 9.4 Age in control group: Mean 48.9, SD 8.6 Duration of illness: >10 years (mean 28.79)	*n =* 12 participants (11 included in the analyses) Modified IPT with dog-assisted *therapy* 12.5 weeks, 45 min x2 per week	*n =* 9 participants (7 included in the analyses) IPT 12.5 weeks, 45 min, x2 per week	LSP (social competence) PANSS (positive, negative and general symptoms) WHOQOL-BREF (quality of life)	RCT (assessment blinded)

5MWT, 5-Meter Walk Test; ACIS, Assessment of Communication and Interaction Skills; BGRS, Budapest Gesture Rating Scale; CHI, Chinese Happiness Inventory; CST, Chair Stand Test; DASS-21, Depression Anxiety Stress Scales Assessment; EQ-5D, EuroQoL-5 Dimensions questionnaire; ILSS, Independent Living Scale Survey; LSP, Living Skills Profile; MHSFS, Mental health-social functioning scale; MoCA, Montreal Cognitive Assessment; PANSS, Positive and Negative Syndrome Scale; QLESQ, Quality of Life Enjoyment and Satisfaction Questionnaire; SAFS, Social adaptive function scale; SANS, Schedule for the Assessment of Negative Symptoms; SHAPS, Snaith-Hamilton Pleasure Scale; SQLS, Subjective Quality of Life Scale; STAI, State-Trait Anxiety Inventory; TUG, Timed Up-and-Go; WHOQOL-BREF, Brief World Health Organization Quality of Life Assessment.

DSM-IV-TR, Diagnostic and Statistical Manual of Mental Disorders-IV-text revision; F, Females; IPT, Integrated psychological treatment; M, Males; N/A, Not applicable; RCT, Randomized controlled trial; SD, Standard deviation.

^a, b^Subgroup data not available. The numbers refer to the whole group, including mood disorders, psychotic disorders, substance use disorders and other disorders.

^c^Reviewer's interpretation based on the following information: 21 took part in the intervention group, 5 dropped out and 3 did not meet eligibility criteria. Nevertheless, Tables 1, 2 in the article are showing n = 21 in the intervention group.

^d^Reviewer's interpretation based on the following information: 15 took part in the control group, 3 dropped out and 2 did not meet eligibility criteria. Nevertheless, Tables 1, 2 in the article are showing n = 15 in the control group.

^e^Measured after session 3, 6 and 10.

The results were presented as significant or non-significant. Significant results were presented with p-values and associated statistics, most commonly averages and standard deviations. As statistical methods varied among the studies and confidence intervals were not stated, it was not possible to select a common effect measure across studies. Substantial heterogeneity regarding interventions and outcomes prohibited meta-analysis. Studies were grouped for narrative synthesis based on outcome measures. Effect sizes were presented in the synthesis for the outcomes where effect size was calculated.

Effect sizes measured by Cohen's *d* were categorized as small for values from 0.2 to 0.49, as medium for values from 0.50 to 0.79, and as large for values above 0.79 (49). Effect sizes measured by SRD were categorized as small for values from 0.11 to 0.27, as medium for values from 0.28 to 0.43, and as large for values above 0.43 (50). The categorization corresponded to the presentation of effect sizes in one of the studies (29). In another study, effect sizes were described by percentage and not presented as small, medium, or large (44). In this SR, the descriptive presentations of effect sizes from the abovementioned study (44) were therefore based on recommendations by Cohen (49).

### 2.4. Risk of bias and quality of evidence

Risk of bias was assessed independently by two reviewers (MT and SS) for each outcome using RoB2 (Risk of Bias 2) tool (51) for RCTs, a specialized version of RoB2 for cluster-randomized trials (52), and ROBINS-I (Risk Of Bias In Non-randomized Studies—of Interventions) (53) for the remaining studies. In addition to assessments related to reporting bias covered under RoB2 and ROBINS-I (bias due to missing data), correlation between trial registers (ClinicalTrials.gov) and published studies were considered with regard to publication bias.

The quality of evidence was assessed independently by two reviewers (EJ and MT) based on guidelines from GRADE (Grading of Recommendations Assessment, Development and Evaluation) handbook (54) and an article regarding imprecision (55). In addition, an article with guidelines regarding quality of evidence in SRs without meta-analyses was used (56). In accordance with GRADE (54), the evidence across studies was graded as high, moderate, low, or very low for each outcome. Risk of bias, publication bias, inconsistency, indirectness, and imprecision were assessed for potential downgrading of the certainty of evidence. While serious limitations may lead to downgrading by one level, very serious limitations may lead to downgrading by two levels. On the other hand, large magnitude of effect may lead to upgrading by one or two levels, while large dose-response gradient and effect-reducing confounders may lead to upgrading by one level.

## 3. Results

### 3.1. Selection of studies

**Figure 1:**
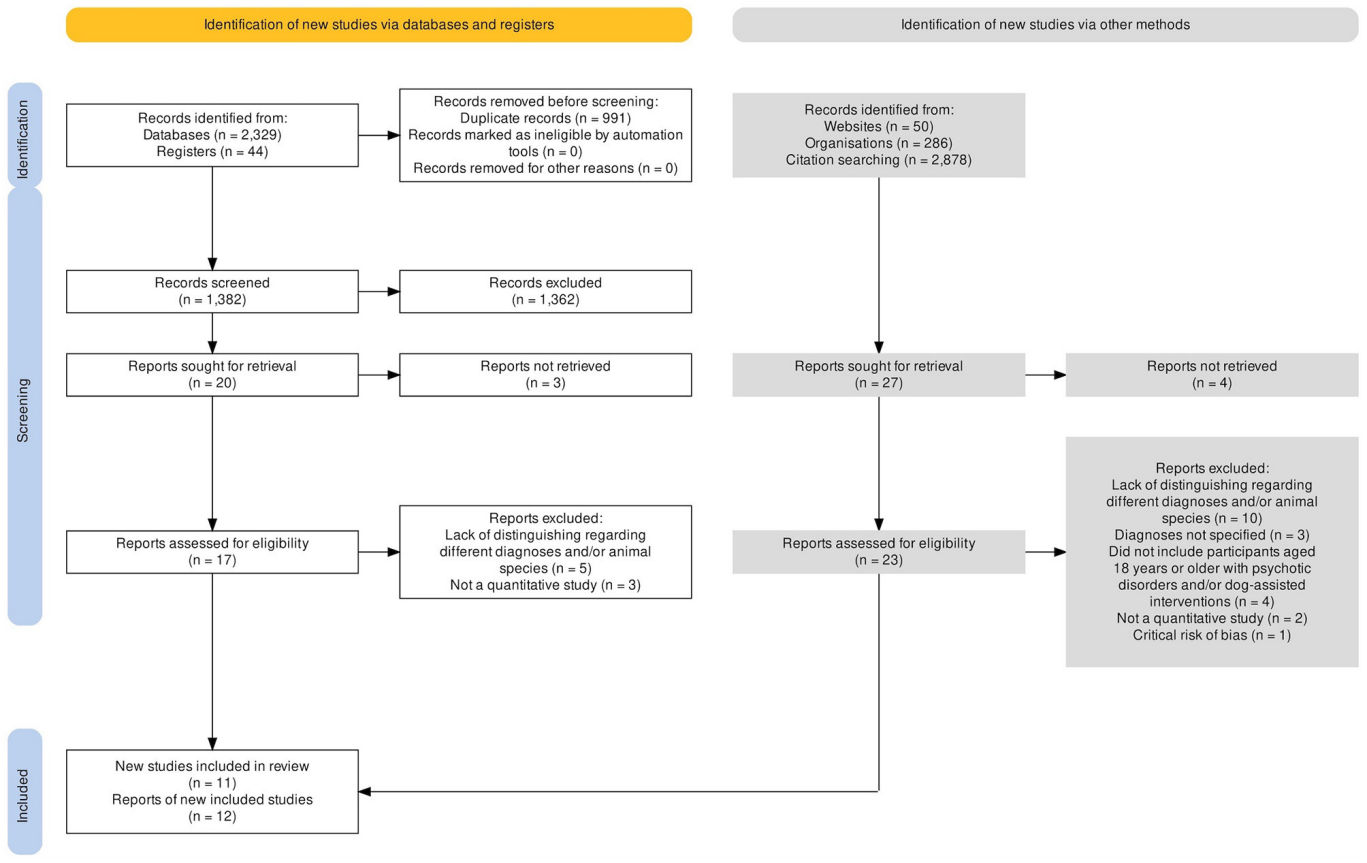
PRISMA flow diagram. Overview of the selection process. The flow diagram was created via a tool in accordance with the PRISMA statement (57).

The main searches retrieved a total of 2,296 records. The total number of identified records was 2,329 after supplementary searches in additional databases. Searches in Google Scholar, in websites of organizations, and citation searches additionally expanded the number of records to 5,587. Details are presented in Figure 1. Nine of the articles from the main database searches met eligibility criteria. Three additional articles published during 2022 and 2023 were included after updated searches in Google Scholar. These articles were also detected through automatic database alerts. No additional articles were included after searches for unpublished literature or through citation searches. At the time of the most updated search, performed 28.05.23 in Google Scholar, no new publications were discovered. This was consistent with simultaneous assessments of the automatic database alerts from the main searches. Summarized, 12 articles, based on 11 studies, met eligibility criteria. An overview of studies excluded after review in full text version, or due to lack of access to full text version, is presented in Supplementary Table 14.

### 3.2. Study characteristics

References and details regarding study characteristics are presented in Table 2. In the 11 eligible studies, a total of 196 participants were included in intervention groups and 179 were included in control groups. The phase of disorder was described as chronic in six of the studies, as acute in two, and not specified in the remaining. All studies included both females and males. The participants were recruited from inpatient settings in eight of the studies, from a residential treatment center in one, from a day-care unit in one, and from both a psychiatric rehabilitation ward and a day-care ward in one. Baseline treatment, which was not stated in all studies, consisted of antipsychotic medications and different psychosocial treatments. In some of the studies, it was stated that all participants received stable antipsychotic treatment (29, 30, 43, 44). Where analyzed, no significant differences were found between the intervention group and the control group regarding antipsychotics (31).

The interventions were described as therapy in nine of the studies, as activity in one, and as interview in one. Four studies were designed as RCTs, one as a controlled pilot study, two as crossover studies, two as pilot/exploratory studies, one as longitudinal, single-blind experimental study, and one as quasi-experimental prospective study. Outcome measures were overall positive and negative symptoms, anhedonia, general psychopathology including isolated measurements of depression, emotional symptoms and anxiety, living skills, social function, social adaptive function, stress, extent of social support, self-determination, self-esteem, global cognitive function, lower body strength, agility, mobility, communication and interaction skills including isolated measurements of non-verbal communication, quality of life, well-being, and patient experience (adherence).

#### 3.2.1. Intervention details

References and further details regarding the interventions are presented in Table 3. The extent of interventions ranged from a single session consisting of 30 minutes to sessions of 50 minutes per week for nine months. Although there was no standardized program across the studies, elements such as physical activity, cognitive activities, and interaction with other participants were common across several of the studies. Specific information regarding certification of therapy dogs was provided in two studies, and information regarding veterinary examinations was provided in three. It was stated that the intervention providers were both educated in psychiatry and had experience or training within AAI in four of the studies. In two of the studies, the intervention was led by a psychiatrist and a social worker, and by a psychologist without further information given. In one of the studies, the intervention was led by researchers, social workers and professional AAT therapists. In four of the studies, it was stated that the intervention providers were researchers and/or handlers without further information given.

**Table 3:** Intervention details, modified version of TIDieR (template for intervention description and replication) (48).

**Study**	**Description of the intervention**	**Aim**	**Description of the dogs**	**Key elements of intervention**	**Intervention provider**	**Modes of delivery**	**Location**	**Duration**
Barker and Dawson, 1998 (47)	Animal-assisted therapy	Investigate effect on anxiety levels	Two therapy dogs meeting hospital policy for AAT (including vaccination, controllability and temperament)	Handler talked generally about dog and encouraged discussion about pets; dog moved freely around interacting/carrying out basic obedience commands.	Dog handlers	Group session	Hospital setting, USA	30 min x1 (one single session)
Calvo et al., 2016 (39)	Animal-assisted therapy	Analyze impact of AAT on symptomatology and quality of life; Evaluate the patient's experience of AAT sessions; Assess stress relief during AAT sessions.	Five therapy dogs experienced in AAT. Physical and behavioral examination by specialists in veterinary behavioral medicine	Establish emotional bond between participant and dogs; walk the dogs; train and play with the dogs Participants worked in pairs at the start of each session	Researcher (unspecified education)	Group session with eight participants in each group, four of the five dogs present	Outdoors, hospital setting, Spain	6 months, 1 h x2 per week
Chen et al., 2021 (29)	Animal-assisted therapy	Evaluate effect of AAT for middle-aged and older patients with schizophrenia, on psychotic symptoms, negative emotions, and well-being.	Four therapy dogs certified by the Professional Animal-Assisted Therapy Association of Taiwan	Warm-up (establishing contact); therapeutic activities (activity for positive emotions, social activity, cognitive activity, physical activity); grooming and feeding; feedback	Certified animal-assisted therapist Occupational therapist (specialized in psychiatric rehabilitation) Certified dog handler	Group sessions with 10 participants in each group	Spacious and quiet classroom, rehabilitation ward/day care ward, Taiwan	12 weeks, 60–65 min x1 per week
Chen et al., 2022 (30)	Same intervention as Chen et al., 2021 (29)	Evaluate the efficacy of AAT for middle-aged patients with schizophrenia on cognition, physical and social functions	Same dogs as Chen et al., 2021 (29)	Same intervention as Chen et al., 2021 (29)	Same intervention providers as Chen et al., 2021 (29)	Same modes of delivery as Chen et al., 2021 (29)	Same location as Chen et al., 2021 (29)	Same duration as Chen et al., 2021 (29)
Chu et al., 2009 (45)	Animal-assisted activity	Examine a program for pet-assisted activity to determine whether such interactions can positively influence the physiological and psychological aspects of patients with schizophrenia	Two dogs, unspecified training	Dogs led in circle around the participants; participants encouraged to interact with dogs and other participants; physical activity	Researchers (unspecified education)	Group sessions with 15 participants	Garden and activity hall, hospital setting, Taiwan	8 weeks, 50 min x1 per week
Kovacs et al., 2004 (40)	Animal-assisted therapy	Evaluate effects of AAT in institutionalized middle-aged patients with schizophrenia with regards to adaptive functioning.	One dog (no further descriptions)	Establish contact as dog went around participants; simple or complex exercises including interaction with other participants; physical activity; grooming and feeding	Psychiatrist Social worker Dog handler	Group sessions with seven participants	Garden and occupational room, social institute, Hungary	9 months, 50 min x1 per week
Kovacs et al., 2006 (41)	Animal-assisted therapy	Examine effects of AAT in chronic schizophrenia to increase non-verbal communication	Two dogs (one participated in the majority of sessions), well-trained, examined by a veterinarian	Warm-up (establishing contact); goal oriented phase with grooming and feeding the dog; specific exercises and interaction with therapy staff and other participants	Psychiatrist, experienced with group therapy and AAT for patients with schizophrenia Co-therapist (psychology student) Dog handler (psychology student)	Group sessions with five participants	Day-care-unit, Hungary	6 months, 50 min x1 per week
Lang et al., 2010 (42)	Dog-assisted interviews	Evaluate effect of dog assisted interviews on state anxiety in patients with schizophrenia	Two therapy dogs that had been working at the unit several months	Level of interaction with dog determined by participants, but physical interaction not allowed	Research assistant (unspecified education)	Group session with seven participants in each group	Quiet room, hospital setting, Germany	30 min x1
Monfort et al., 2022 (31)	Dog-assisted therapy	Evaluate the AAT-efficacy for patients with dual diagnosis (schizophrenia-spectrum disorders and substance-use disorders) in residential treatment with regard to positive symptoms, negative symptoms, general psychopathology and functionality	A therapy dog	Presentation and greeting, interaction with the dogs and other participants, grooming, information regarding canine behavior, sharing experiences	Psychologist Social educator AAT technician Dog trainer	Groups of maximum 10 participants in each	Residential setting, Spain	Maximum of 12 weeks (10 sessions), 45 min per week
Nathans-Barel et al., 2005 (43)	Animal-assisted therapy	Examine beneficial effects of animal-assisted therapy on anhedonia in chronic schizophrenia	One dog approved by a veterinarian	Participants could choose from different activities with the dog including talking, making contact, petting, feeding, cleaning, teaching, taking the dog for a walk	Psychology student qualified as animal trainer and experienced with animal assisted interventions	Group sessions with 10 participants	Hospital setting, Israel	10 weeks, 60 min x1 per week
Shih et al., 2023 (32)	Animal-assisted therapy	Evaluate effectiveness of AAT on social interactions and quality of life for patients with schizophrenia during COVID-19	Two service dogs, minimum 3 months of training	Building relationships with dogs and other participants; brief interaction (including simple instructions, walking and feeding); deeper interaction (including grooming and group activities)	Researchers Social workers Two professional AAT-therapists (minimum 6 months of training/courses)	Group sessions	The reception hall, hospital setting, Taiwan	12 weeks, 1 h x1 per week
Villalta-Gil et al., 2009 (44)	Dog-assisted therapy	Assess effectiveness of trained therapy dog in institutionalized patients with chronic schizophrenia	One certified therapy dog	Cognitive activities; interaction with the dogs and other participants	Psychologist Dog handler	Group sessions with four participants in each group	Hospital setting, Spain	12.5 weeks, 45 min x2 per week

AAT, Animal-assisted therapy.

### 3.3. Results of individual studies

Significant results were defined as *p* ≤ 0.05 or *p* < 0.05 by the included studies. The results from each study are presented in Table 4.

**Table 4:** Results of individual studies.

	**Between groups**	**Within intervention groups**	**Within control groups**
Barker and Dawson, 1998 (47)	**STAI** (change) Anxiety: I = C	*Stated as mean (SD)* **STAI** (change) Anxiety: 5.77 (13.72), *p < * 0.006	**STAI** Anxiety: NS
Calvo et al., 2016 (39)	*Stated as mean (SD)* **PANSS** (change/posttreatment) Positive: I = C Negative: I = C General: I = C **EQ-5D** (change/posttreatment) Total score: I = C Health today 12m: I = C Mobility: I = C Pain/discomfort: I = C Health state today: I = C Anxiety/depression: I = C Daily activities: I = C Personal care: I = C **Adherence** Overall: I > C: 92.9% (4.7) vs. 61.2% (24.8), *p =* 0.001 Specific functional rehabilitation interventions: AAT vs. art therapy: I > C, *p =* 0.01 AAT vs. gymnastics: I > C, *p =* 0.01 AAT vs. psychodynamic therapy: N/A AAT vs. group sport: N/A	*Stated as mean (SD)* **PANSS** (change) Positive: 5.28 (4.78), *p =* 0.001 Negative: 5.64 (8.19), *p =* 0.022 General: 10.00 (8.70), *p =* 0.001 **EQ-5D** Total score: NS Health today 12m^a^: NS Mobility: NS Pain/discomfort: NS Health state today: NS Anxiety/depression: NS Daily activities: NS Personal care: NS **Stress relief** Salivary cortisol^b^: Decrease, *p < * 0.05 Salivary alpha-amylase^*a*^: Change, NS	*Stated as mean (SD)* **PANSS** (change) Positive: 7.87 (4.29), *p =* 0.001 Negative: NS General: 12.63 (13.57), *p =* 0.033 **EQ-5D** Total score: NS Health today 12m: NS Mobility: NS Pain/discomfort: NS Health state today: NS Anxiety/depression: NS Daily activities: NS Personal care: NS
Chen et al., 2021 (29)	*Stated as median* **PANSS** Total (change): I > C: −1.0 vs. 0, *p =* 0.001, SRD 0.15 Total (posttreatment): I = C Positive (change): I > C: −3 vs. 0, *p < * 0.001 Positive (posttreatment): I = C Negative (change): I > C: −3 vs. 0, *p < * 0.001, SRD 0.50 Negative (posttreatment): I = C General (change): I > C: −7 vs. 0, *p < * 0.001, SRD 0.20 General (posttreatment): I = C **DASS-21** Total (change/posttreatment): I = C Stress (change): I > C, −1.0 vs. 1.5, *p =* 0.012, SRD 0.15 Stress (posttreatment): I = C Anxiety (change/posttreatment): I = C Depression (change/posttreatment): I = C **CHI** Well-being (change/posttreatment): I = C	N/A	N/A
Chen et al., 2022 (30)	*Stated as median* **MoCa** Global cognitive function (change): I = C Global cognitive function (posttreatment): I = C **CST** Lower body strength (change) I > C, 0.50 vs. −1.00, *p =* 0.007 Lower body strength (posttreatment): I = C	N/A	N/A
	**TUG** Agility (change): I = C Agility (posttreatment): I = C **5MWT** Mobility (change): I = C Mobility (posttreatment): I = C **ACIS** Communication and interaction skills (change): I > C, 5.00 vs. 0.50, *p < * 0.001 Communication and interaction skills (posttreatment): I > C, 71.50 vs. 65.00, *p =* 0.003		
Chu et al., 2009 (45)	*Stated as mean* **Questionnaire** (change) Self-esteem: I > C 6.03 vs. −0.19, *p =* 0.025 Self-determination: I > C: 5.87 vs. −0.21, *p =* 0.020 Social support: I = C Positive symptoms: I > C: −6.42 vs. 0.69, *p =* 0.005 Negative symptoms: I = C Emotional symptoms: I > C: −5.62 vs. 0.13, *p =* 0.048	N/A	N/A
Kovacs et al., 2004 (40)	N/A	*Stated as mean (SD)* **ILSS, degree of behavioral problems** Domestic activities: 0.97 (0.93) to 0.37 (0.58), *p =* 0.01 Health: 0.90 (0.77) to 0.33 (0.66), *p =* 0.02 Leisure: NS Money management: NS Transportation: NS Eating: NS Grooming: NS **ILSS, frequency of occurrence of behaviors** Domestic activities: 2.06 (1.18) to 3.26 (0.74), *p =* 0.01 Health: 2.71 (0.48) to 3.40 (0.24), *p =* 0.01 Leisure: NS Money management: NS Transportation: NS Eating: NS Grooming: NS	N/A
Kovacs et al., 2006 (41)	N/A	**BGRS** Nonverbal communication: Significance was not investigated	N/A
Lang et al., 2010 (42)	*Stated as mean (SD)* **STAI** (change) Anxiety: I > C: 45.9 (11.8) to 35.6 (11.0) vs. 42.4 (11.1) to 40.1 (10.5), *p < * 0.0001	N/A	N/A
Monfort et al., 2022 (31)	*Stated as mean* **PANSS** (posttreatment) Positive: I > C, 27.81, *p =* 0.002 Negative: I = C General: I = C **LSP-20** (posttreatment) Life skills (total): I > C, 20.44, *p =* 0.001	N/A	N/A
Nathans-Barel et al., 2005 (43)	*Stated as mean (SD)* **SHAPS** (posttreatment) Hedonic tone: I > C, 3.44 (0.40) vs. 3.12 (0.34), *p =* 0.02 **QLESQ** (posttreatment) Leisure time activities: I > C: 3.75 (0.91) vs. 3.47 (0.71), *p =* 0.01 Physical health: I = CSubjective feelings: I = C Social relationships: I = C General activities: I = C Work: I = C Household duties: I = C Medication satisfaction: I = C School/course work: I = C Life satisfaction and enjoyment: I = C **SQLS** (posttreatment) Psychological: I = C Motivation: I = C Side effects: I = C **PANSS** (posttreatment) Total: I = C Positive: I = C **SANS** (posttreatment) Negative symptoms: I = C	N/A	N/A
Shih et al., 2023 (32)	*Stated as B (SE) group x time (reference control group x baseline)* **MHSFS** Social function (T2): I > C, B (SE) = 1.16, *p =* 0.043 Social function (T3): I < C, B (SE) = −5.37, *p =* 0.037 **SAFS** Social adaptive function (T2): I = C Social adaptive function (T3): I = C **WHOQOL** Quality of life (T2): I > C, B (SE) = 4.44, *p =* 0.044Quality of life (T3): I > C, B (SE) = 11.06, *p =* 0.007	*Stated as mean (SD)* **MHSFS** Social function (change *t*^1^): 50.56 (11.89) to 52.80 (11.93), *p < * 0.01 Social function (change, *t*^2^): NS **SAFS** Social adaptive function (change *t*^1^): 11.56 (7.66) to 9.87 (7.69), *p < * 0.01 Social adaptive function (change, *t*^2^): NS **WHOQOL** Quality of life (change *t*^1^): 79.33 (13.40) to 86.42 (17.98), *p < * 0.01 Quality of life (change, *t*^2^): 79.33 (13.40) to 86.64 (15.92), *p < * 0.01	*Stated as mean (SD)* **MHSFS** Social function (change, *t*^1^): 54.09 (13.80) to 55.18 (14.34), *p < * 0.05Social function (change, *t*^2^): NS **SAFS** Social adaptive function (change *t*^1^): 11.87 (7.67) to 10.51 (8.21), *p < * 0.05 Social adaptive function (change, *t*^2^): 11.87 (7.67) to 10.16 (7.46), *p < * 0.05 **WHOQOL** Quality of life (change *t*^1^): NS Quality of life (change, *t*^2^): NS
Villalta-Gil et al., 2009 (44)	**LSP** (posttreatment) Self-care: I = C Social behavior: I = C Social contact: I = C Non-personal social behavior: I = C Autonomous life: I = C **PANSS** (posttreatment) Total: I = C Positive: I = C Negative: I = CGeneral: I = C **WHOQOL-BREF** (posttreatment) Physical health: I = C Psychological: I = C Social relationships: I = C Environment: I = C	*Stated as mean (SD)* **LSP** Self-care: NS Social behavior: NS Social contact: 13.67 (2.67) to 18.00 (4.40), *p < * 0.05, Cohen's *d -*1.19 Non-personal social behavior: 22.50 (1.38) to 20.55 (2.94), *p < * 0.05, Cohen's *d* 0.85 Autonomous life: NS **PANSS** Total: 88.25 (12.17) to 73.64 (18.69), *p < * 0.05 Positive: 20.83 (5.46) to 15.64 (4.03), *p < * 0.01, Cohen's *d* 1.08 Negative: 28.92 (5.25) to 19.36 (6.34), *p < * 0.01, Cohen's *d* 1.64 General: NS **WHOQOL-BREF** Physical health: NS Psychological: NS Social relationships: 2.08 (0.79) to 2.85 (0.56), *p < * 0.05, Cohen's *d* −1.12 Environment: NS	*Stated as mean (SD)* **LSP** Self-care: NSSocial behavior: NS Social contact: NS Non-personal social behavior: NS Autonomous life: NS **PANSS** Total: 86.22 (10.03) to 61.83 (12.69), *p < * 0.05 Positive: 22.67 (7.71) to 17.00 (6.07), *p < * 0.05, Cohen's *d* 0.82 Negative: NS General: 38.11 (5.82) to 28.50 (5.58), *p < * 0.05, Cohen's *d* 1.69 **WHOQOL-BREF** Physical health: NS Psychological: NS Social relationships: NS Environment: NS

5MWT, 5-Meter Walk Test; ACIS, Assessment of Communication and Interaction Skills; BGRS, Budapest Gesture Rating Scale; CHI, Chinese Happiness Inventory; CST, Chair Stand Test; DASS-21, Depression Anxiety Stress Scales Assessment; EQ-5D, EuroQoL-5 Dimensions questionnaire; ILSS, Independent Living Scale Survey; LSP, Living Skills Profile; MHSFS, Mental health-social functioning scale; MoCA, Montreal Cognitive Assessment; PANSS, Positive and Negative Syndrome Scale; QLESQ, Quality of Life Enjoyment and Satisfaction Questionnaire; SAFS, Social adaptive function scale; SANS, Schedule for the Assessment of Negative Symptoms; SHAPS, Snaith-Hamilton Pleasure Scale; SQLS, Subjective Quality of Life Scale; STAI, State-Trait Anxiety Inventory; TUG, Timed Up-and-Go; WHOQOL-BREF, Brief World Health Organization Quality of Life Assessment.

AAT, Animal assisted therapy; C, Control group; I, Intervention group; N/A, Not applicable; NS, Not significant; SD, standard deviation; SE, standard Error; SRD, Success rate difference; *t*^1^, Change from baseline to posttreatment; *t*^2^, Change from baseline to follow-up 3 months after intervention; T2, Posttreatment; T3, 5 months follow-up; v.s., Vs.

^a^Significant before Bonferroni correction, non-significant after Bonferroni correction.

^b^Only measured within the intervention group.

### 3.4. Synthesis

#### 3.4.1. Positive and negative symptoms

Three studies showed significant improvement for the intervention group compared with the control group for positive symptoms (29, 31, 45). The effect size in one of the studies was small (29). Two studies showed significant improvement both within the intervention group and within the control group, and no significant differences were found between the groups (39, 44). The effect sizes within both groups in one of the studies were large (44). One study found no significant difference between the groups, and significance within the groups was not stated (43).

With regard to negative symptoms in general, one study showed significant improvement, with large effect size for the intervention group compared with the control group (29). One study showed significant improvement for the intervention group compared with the control group for anhedonia (43). Two studies showed significant improvement within the intervention groups, and not within the control groups, for negative symptoms in general (39, 44). The effect size was large in one of the studies (44). The differences between the groups were not significant. Two studies found no significant differences between the groups. Significance within the groups were not stated (43, 45). In one of the studies, the groups were described as “not comparable” due to significant pre-intervention differences. There were no significant differences at the end of the intervention (31).

#### 3.4.2. General psychopathology including isolated assessments of emotional symptoms, anxiety and depressive symptoms

With regard to general psychopathology, one study showed significant improvement, with small effect size, for the intervention group compared with the control group (29). One study showed significant improvement both within the intervention group and within the control group. No significant difference was found between the groups (39). One study showed significant improvement, with large effect size, within the control group, and no significant change within the intervention group. The difference between the groups was not significant (44). One study showed no significant differences between the intervention group and the control group. Significance within the groups was not stated (31).

One study showed significant improvement for the intervention group compared with the control group for emotional symptoms (45). With regard to anxiety, one study showed significant improvement for the intervention group compared with the control group (42). Furthermore, one study showed significant improvement within the intervention group, and not within the control group. There was no significant difference between the groups (47). One study found no significant improvement for anxiety and depressive symptoms. Significance within the groups was not stated (29).

#### 3.4.3. Living skills, stress, self-esteem, self-determination, social contact and cognition

One study showed significant improvement for living skills for the intervention group compared with the control group (31). One study, not including a control group, showed significant improvement within the intervention group for independent living skills related to domestic activities and health. There were no significant changes for several other aspects of living skills in the same study (40). Another study showed significant improvement within the intervention group, with large effect size, for living skills related to social contact. No significant improvement was observed within the control group. The difference between the groups was not significant. Furthermore, the study showed a significant deterioration, with large effect size, for non-personal social behavior within the intervention group. There was no significant change in non-personal social behavior within the control group. The difference between the groups was not significant. The same study found no significant change for other domains of living skills (44).

One study showed significant improvement, with small effect size, for the intervention group compared with the control group for stress (29). Another study showed significant improvement within the intervention group for change in cortisol levels. No significance was found for change in alpha-amylase. These markers were not investigated within the control group (39). With regard to self-esteem and self-determination, one study showed significant improvement for the intervention group compared with the control group. The same study found no significant difference between the groups for extent of social support. Significance within the groups was not stated (45). One study showed significant improvement for the intervention group compared with the control group for social function measured at the end of the intervention period. However, at 3 months follow-up, results were opposite, with significant improvement for the control group compared with the intervention group. Regarding social adaptive function, the same study showed no significant change between the groups. There were significant improvements both within the intervention group and within the control group at post-intervention. Nevertheless, only the control group had significant improvement at 3 months follow-up

One study showed significant improvement for the intervention group compared with the control group for communication and interaction skills (30). One study, without a control group, indicated improvement within the intervention group for use of space during communication and partial improvement for anatomy of movement, dynamics of movements and regulating movements. Calculation of significance was not performed (41). One study found no significant difference between the intervention group and the control group in global cognitive function (30).

#### 3.4.4. Physical performance

One study showed significant improvement for the intervention group compared with the control group for lower body strength. No significant changes between the groups were found for agility and mobility measured by the same study. Significance within the groups was not stated (30).

#### 3.4.5. Quality of life and wellbeing

One study showed significant improvement for the intervention group compared with the control group for quality of life in general, both with regard to post-treatment and 3 months follow-up compared with baseline (32). One study showed significant improvement for the intervention group compared with the control group for quality of life related to utilization of leisure time. There was no significant difference between the groups for a range of other factors of quality of life. Significance within the groups was not stated (43). Another study showed significant improvement, with large effect size, within the intervention group for quality of life related to social relationships. There was no significant findings within the control group. The difference between the groups was not significant. Further, there were no significant findings in this study for quality of life related to other factors (44). One study showed significant improvement before Bonferroni correction within the intervention group, and not within the control group, for quality of life related to general health. The difference between the groups was not significant. There were no significant findings for other domains of quality of life in this study (39). With regard to well-being, one study found no significant difference between the intervention group and the control group. Significance within the groups were not stated (29).

#### 3.4.6. Adherence

One study showed significantly higher adherence, 93% compared with 61%, for the intervention group compared with the control group (39). The reasons for non-adherence within the intervention group were mostly related to family or health issues. Adherence, measured as proportion of attended sessions, was not stated as an outcome measure in the other studies. However, lost to follow-up was documented in most of the studies (presented in Table 2).

### 3.5. Risk of bias

**Figure 2:**
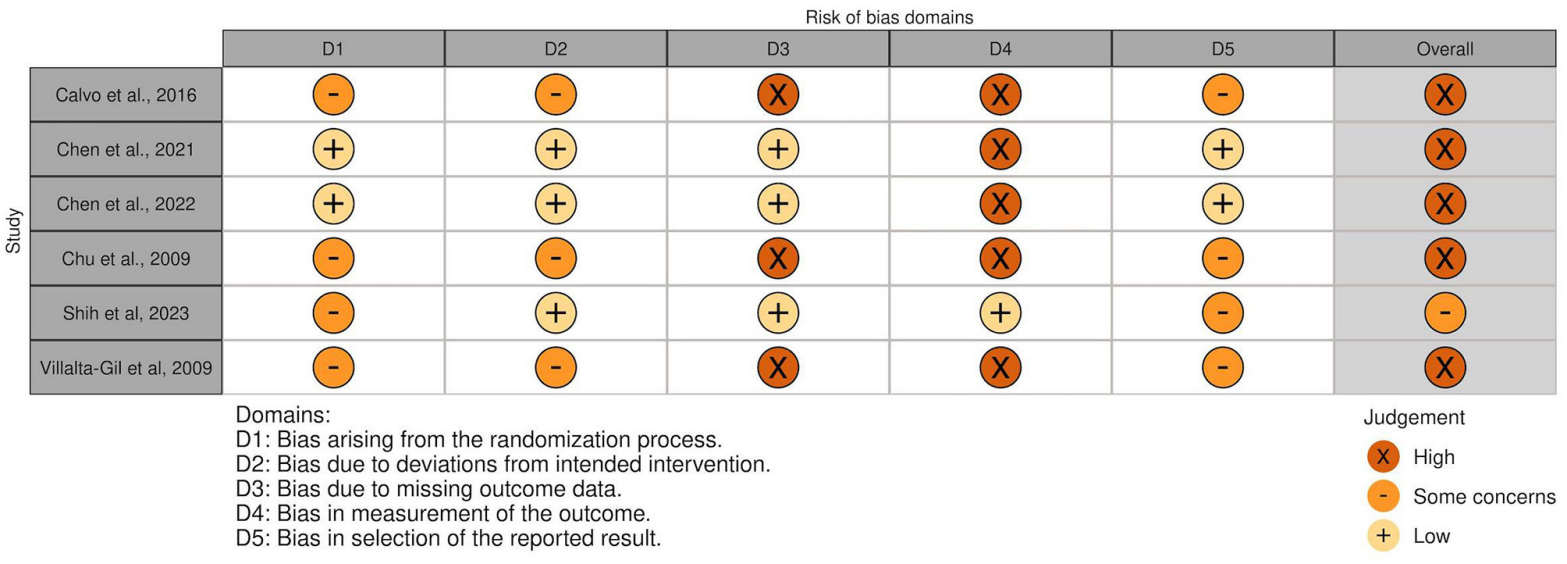
Risk of bias in RCTs and cluster-randomized trials. The highest overall risk for each study is presented as most of the outcomes within each study were associated with the same risk. An exception applied to domain 4. For this domain, alpha-amylase, cortisol and adherence measured by Calvo et al. (39) were associated with low risk. The same applied to agility, lower body strength, and mobility measured by Chen et al., (30), which resulted in overall low risk of bias for these three outcomes. Social competence measued by Villalta-Gil et al. (44) was associated with some concerns for the abovementioned domain. The figure was created via “Risk-of-bias VISualization”-tool (58).

**Figure 3:**
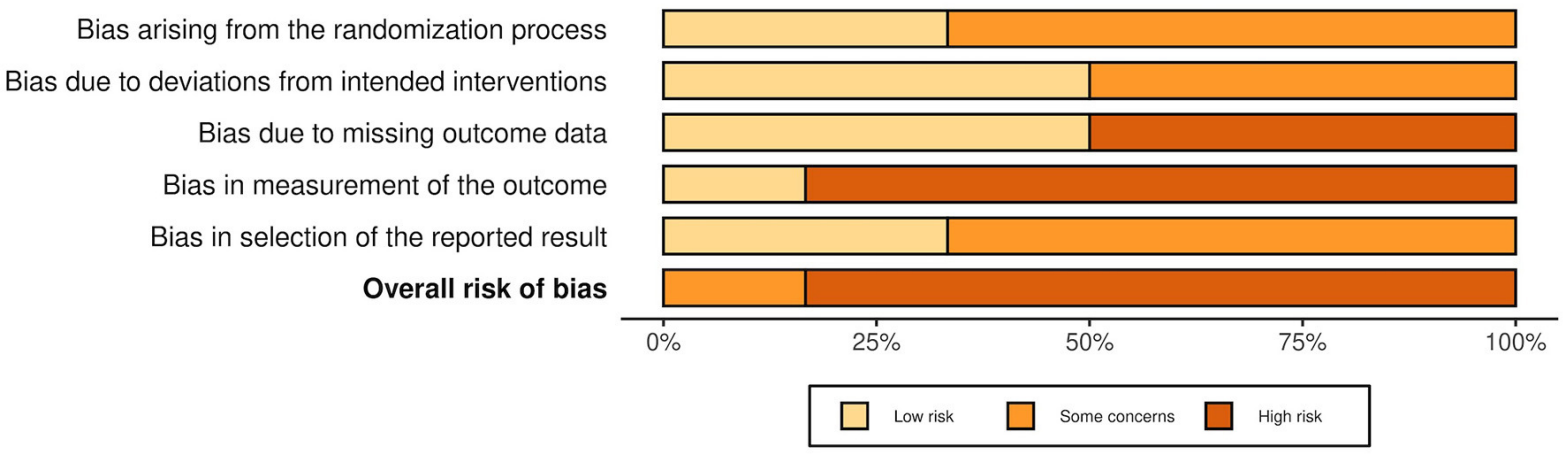
Summarized risk of bias in RCTs and cluster-randomized trials. Summarized risk of bias across studies. The figure was created via “Risk-of-bias VISualization”-tool (58).

**Figure 4:**
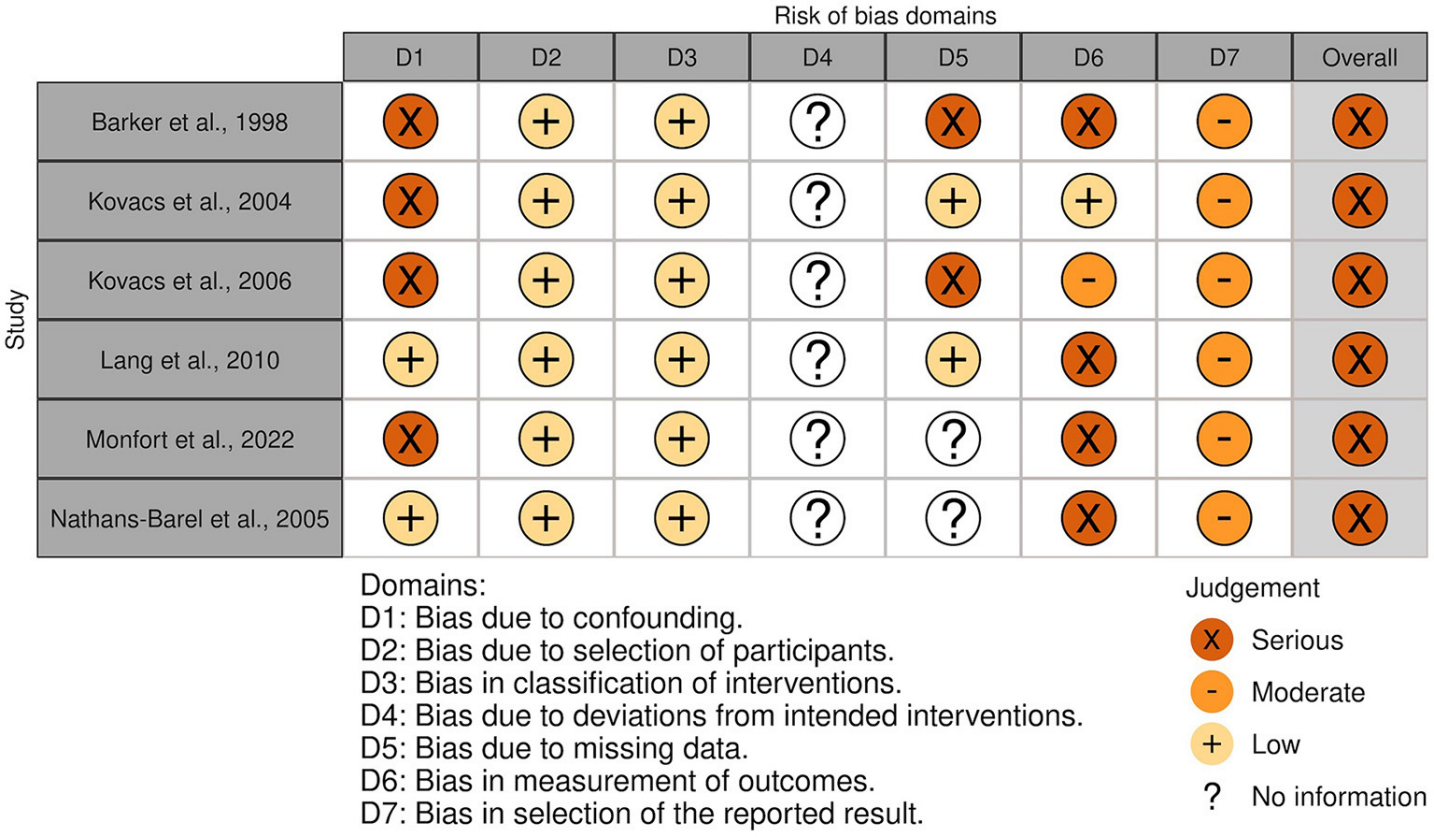
Risk of bias in NRSs. The highest overall risk for each study is presented as most of the outcomes within each study were associated with the same risk. An exception applied to domain 6. For this domain, life skills measured by Monfort et al. (31) were associated with low risk. The figure was created via “Risk-of-bias VISualization”-tool (58).

**Figure 5:**
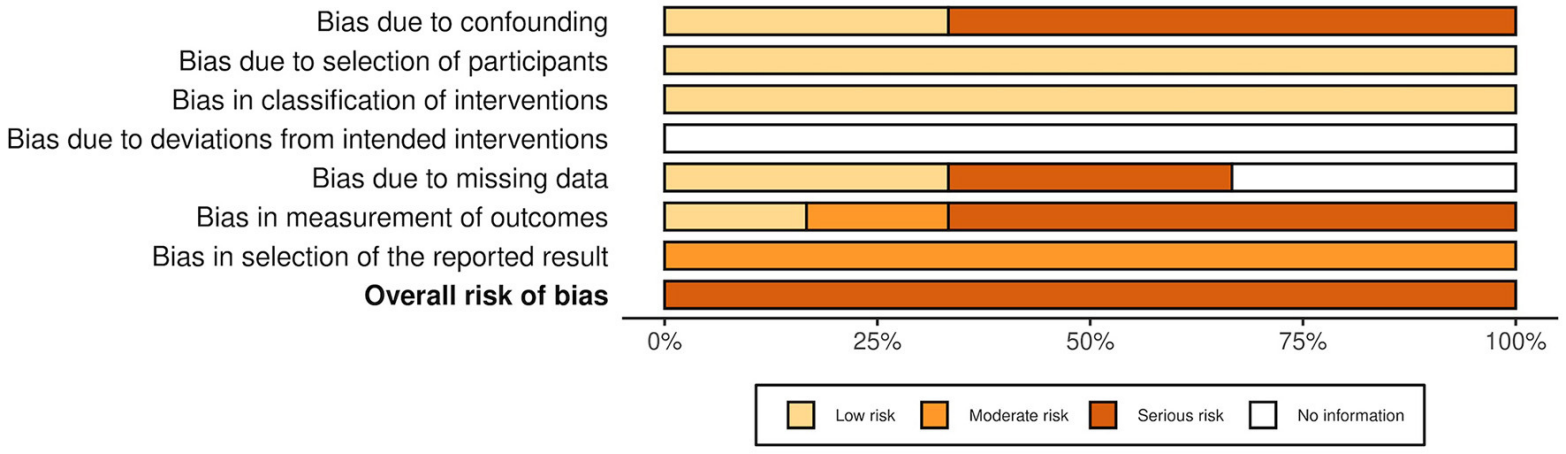
Summarized risk of bias in NRSs. Summarized risk of bias across studies. The figure was created via “Risk-of-bias VISualization”-tool (58).

The overall risk of bias was associated with some concerns for all outcomes in the cluster-randomized trial (32) and with high risk for most of the outcomes in the RCTs (29, 30, 39, 44, 45). Agility, lower body strength, and mobility measured by Chen et al. (30) were outcome measures with low risk of bias. For the non-randomized studies (NRS) (31, 40–43, 47), the overall risk of bias was categorized as serious for all outcomes. An overview is presented in Figures 2–5. One NRS was excluded due to critical risk of bias (59). The material included both studies with significant and non-significant results. No findings were made of studies reported in trial registers not published. Consequently, the risk of publication bias on the field was considered low. Nevertheless, most of the included studies lacked protocols.

### 3.6. Certainty of evidence

Inconsistency, indirectness, imprecision, and risk of bias were factors that led to downgrading of the quality. It was not possible to upgrade the quality due to serious and very serious limitations. The quality of evidence was considered low for agility, lower body strength, and mobility. For the rest of the outcomes, the quality of evidence was considered very low. Details concerning the assessments are presented in Supplementary Tables 16–38.

## 4. Discussion

In this SR, exclusively including studies with isolated results for adults diagnosed with schizophrenia and related disorders, numerous outcomes of DAI were examined. Both significant improvement and non-significant findings for the intervention groups compared with the control groups were reported for general symptoms, positive symptoms, negative symptoms, anxiety, living skills and quality of life. Significant improvement in the intervention groups compared with the control groups was also described for emotional symptoms, stress, self-esteem, self-determination, social function, communication and interaction skills, lower body strength, and adherence, but each of these outcome measures was only examined in single studies.

Within intervention groups, significant improvement was in addition described for salivary cortisol and social adaptive function, also examined in single studies. One study indicated significant deterioration of non-personal social behavior within the intervention group. Specific investigation of wellbeing, depression, agility, mobility, global cognitive function, alpha-amylase, and extent of social support was performed in single studies, and these outcome measures had no significant changes. Significance for non-verbal communication was not stated.

Heterogeneity in study design and lack of statistical calculations complicated the assessment of study outcomes. Therefore, we considered the findings in relation to factors that may have influenced results. Positive symptom score was the outcome measure with the most convincing findings. Significant improvement was demonstrated in several studies, but it should be noted that this was the outcome measure investigated by most studies. Findings concerning negative symptoms, the second most investigated outcome measure, were more divergent. Overall, inconsistent results were reported for the majority of outcome measures examined by more than one study. In the following sections, we highlight some potential explanations.

Importantly, a substantial difference across the studies was related to the content of the control groups. As an example, the studies by Villalta-Gil et al. (44) and Calvo et al. (39) included specific treatment programs focusing on psychosocial aspects with and without DAT. On the other hand, Chu et al. (45) compared AAA with treatment as usual. While significant improvement for several outcomes occurred within the groups in the first two studies, results from the latter contrasted the two abovementioned studies with a substantially larger degree of significant effects of active treatment. Active intervention also occurred in control groups in other studies—e.g. therapeutic recreation (music, art and education) as comparator in the study by Barker et al. (47). No significant changes for anxiety were seen between the groups in this study. The findings were contrasted by results in the study by Lang et al. (42) where the presence of a dog seemed to be the only difference between the groups (42). The findings suggest that the content in the control group may contribute largely to the heterogeneity of results across studies. As there are many uncertainties related to the effects of components only presented in DAI, a specific recommendation for future research is to conduct component studies. This suggestion is in accordance with a SR regarding factors of AAI (60) and a study on the role of common factors in psychotherapy (61).

A second issue contributing to lack of significant results may be related to other aspects of study design: low numbers of participants or short duration of interventions. As an example, the study by Shih et al. (32) showed significant improvement for quality of life between the groups. Increased overall quality of life was not shown in the other studies, neither between the groups or within the groups. The study by Shih et al. (32) stood out with a higher number of participants. Similarly, in one study showing significant improvement for the intervention group compared with the control group for both positive and negative symptoms, the population consisted of 40 participants (29). For the four studies with non-significant changes for negative symptoms, the samples were smaller with 18 to 23 participants included in analyses (31, 39, 43, 44).

Finally, study participant heterogeneity is likely to influence results on many levels. It is conceivable that treatment effects of different psychosocial interventions will vary based on individual characteristics such as symptom burden and preferences. Conditions reflecting symptom burden and level of functioning are reflected in the included trials: participants in the studies with significant changes between the groups for positive symptoms measured by PANSS, were recruited from a psychiatric rehabilitation ward (29), from a day care center (29) and from a residential center (31), whereas participants in the studies with non-significant changes were hospitalized (39, 43, 44). Nevertheless, analyses of 27 hospitalized participants showed significant changes between the groups for positive symptoms, and not for negative symptoms, measured by a questionnaire (45). Recommendations for further research include dividing participants into subpopulations as well as investigating whether the effectiveness of AAI varies based on severity of symptoms, demanding a relatively high number of participants.

Diverging results have also been documented in previous SRs on related topics. As an example, a SR on dog presence and therapeutic alliance stated that half of the studies showed effect. Heterogeneity in study characteristics was described as an important limitation (62). Some of the results in our SR, however, contrasted earlier findings. In the SR by Hawkins et al. (25) including RCTs on AAI in general for individuals diagnosed with schizophrenia and related disorders, no improvement regarding quality of life was reported. One of the studies included in our SR indicated the opposite (32). This underpins that the field is under continuous development.

One of the purposes of this SR was to examine somatic effects, which were directly assessed in some of the studies through examination of physical skills and measurements of biochemical markers (30, 39). However, heart rate, blood pressure, HbA1c and lipid levels have not yet been investigated for adults diagnosed with schizophrenia and related disorders participating in DAI. This will be of importance as the population is at high risk of metabolic syndrome (63). Beneficial effects on cardiovascular risk factors have been associated with dog companionship or therapy for varied populations, but it is stated that further research is needed (64).

Another outcome measure especially relevant for further investigation is motivation. No significant changes were found for motivation related to treatment as a measure of quality of life, but this was only investigated in one study (43). Adherence can also function as an indicator of motivation. The adherence was significantly higher in the intervention group compared with the control group in one study (39). However, the results remain inconclusive. In addition to stating of reasons for non-adherence and lost to follow-up, validated instruments such as IMI-SR (65) may provide valuable information in further studies.

Significant worsening of non-personal social behavior within the intervention group was reported in one study (44). According to the authors, the intervention was not directed at these aspects and a similar trend was seen within the control group. Overall, prevention of negative consequences should have high priority. Examination by specialists in veterinary behavioral medicine was one of the preventive interventions described in one of the studies (39). Another example was inclusion of another dog in the later stages to reduce anxiety and grief due to removal of the dog at the end of the study (41). In a SR specifically addressing the benefits and risks associated with AAI, allergies, infections and accidents were described as the major risk factors. It was stated that these factors were outweighed by benefits (14). Animal welfare was only mentioned specifically by one of the included studies in our SR (39). Methods for overall safety, prevention of negative consequences and welfare for both participants and animals are of importance to describe in further articles. Development of interventions must be performed in accordance with guidelines for safety and welfare, for example from IAHAIO (13). A specific recommendation for further research is to describe evaluations regarding signs of stress in the participating dogs. In addition, a predetermined plan of action in case of negative consequences is of importance to include.

In addition to abovementioned limitations regarding consistency, a low number of participants led to imprecision. Furthermore, three outcome measures were associated with indirectness due to surrogate measures or use of inappropriate measurement methods. Risk of bias was categorized as high or serious for most of the included studies. Some factors that entailed the risk were missing outcome data, deficient blinding of personnel who assessed the measurements and risk of confounding. However, some of the factors causing risk of bias could not be avoided due to the nature of the interventions.

Exclusion of articles written in other languages than English and Scandinavian led to risk of selection bias in the review process. Lack of access to potentially relevant studies may also have caused bias. Due to lack of variables, such as confidence intervals, findings were reported as significant or non-significant. Such reporting is against the principles of Cochrane, and it must be emphasized that lack of evidence is not the same as lack of effect (66). Therefore, both significant and non-significant findings must be interpreted with caution.

Although inclusion of other designs than RCTs led to lower quality of evidence, these studies proved valuable in this SR through presentation of outcome measures not synthesized in a previous SR (25). Furthermore, inclusion of recent published studies led to novel insight on the topic. Isolated assessments of anhedonia, social function (including social adaptive function), communication and interaction skills (including non-verbal communication), lower body strength, mobility, agility and cognitive function, were among the outcome measures that expanded the knowledge. Specific examination of dog-assisted interventions increased the directness and complemented more general reviews on related topics.

Summarized, the findings suggest that DAI may have an effect on a range of symptoms and features associated with severe psychotic disorders. However, the findings must be interpreted with caution. Due to several knowledge gaps, it is challenging to state specific implications for policy and practice. The trade-off regarding potential benefits and potential harms are important. Based on available data, we consider the potential benefits of DAI to outweigh risk of harmful effects, given that all required precautions are taken. A potential negative consequence was described by only one of the included studies, and the causality of the finding remained uncertain (44). A specific implication for practice, which must be emphasized, is the necessity of development and implementation of interventions in accordance with guidelines for safety and welfare. The lack of data regarding animal welfare assessments is considerable, and the area is overall described as under-researched (67). In addition to the specific recommendations for further research presented in the paragraphs above and in Supplementary Table 39, reduction of bias and increase of quality will be essential. Accordingly, there is a specific need for carefully designed RCTs. This is particularly justified by the risk of confounding associated with NRS.

## 5. Conclusion

The included studies indicate potential effects of dog-assisted interventions for adults diagnosed with schizophrenia and related disorders, mostly beneficial. However, the results must be interpreted with caution due to methodological limitations such as low number of participants, heterogeneity among study design and included participants, and risk of bias. Findings of both significant and non-significant results are in accordance with reviews on animal-assisted interventions in general. Importantly, inclusion of several study designs and novel trials enabled synthesizing of outcome measures not covered by previous reviews. Some of the results, such as significant improvement for quality of life, contrast earlier findings. This underpins that the field is under continuous development, and further examination of causality is warranted. Recommendations for future research include factors such as calculation of effect sizes, development of more standardized programs, and investigation of effects related to motivation and somatic effects.

## Data availability statement

The original contributions presented in the study are included in the article/Supplementary material, further inquiries can be directed to the corresponding author.

## Author contributions

MT designed this systematic review with contributions from all co-authors, wrote the original draft, and all authors participated in revision. EJ and MT extracted data and graded the quality. MT and SS assessed risk of bias. All authors participated in screening and selection of articles and approved the final manuscript.
