# Supplementary material for: Dog-assisted interventions for adults diagnosed with schizophrenia and related disorders: a systematic review
Source: Front Psychiatry. 2023 Jun 23;14:1192075. doi: 10.3389/fpsyt.2023.1192075 (PMC10326428; doi:10.3389/fpsyt.2023.1192075)
Supplement: Supplementary file 1 [file Data_Sheet_1.PDF]

Supplementary Material

1. PRISMA flow diagram 2
2. Search strategies 4
2.1. Development of search strategies 4
2.2. Main searches 5
2.3. Supplementary searches 8
3. Excluded articles 9
4. Data elements 11
5. Quality assessments 11
5.1. General symptomatology 12
5.2. Positive symptoms 12
5.3. Negative symptoms 13
5.4. Anhedonia 13
5.5. Emotional symptoms 13
5.6. Depression 14
5.7. Anxiety 14
5.8. Stress 14
5.9. Non-verbal communication 15
5.10. Self-esteem 15
5.11. Self-determination 15
5.12. Living skills 16
5.13. Social function 16
5.14. Social adaptive function 16
5.15. Communication and interaction skills 17
5.16. Extent of social support 17
5.17. Global cognitive function 17
5.18. Lower body strength 18
5.19. Agility 18
5.20. Mobility 18
5.21. Quality of life 18
5.22. Well-being 19
5.23. Patient experience (adherence) 19
6. Recommendations for further research 20

1. PRISMA flow diagram

Supplementary table 1: PRISMA flow diagram (1)

Section and Topic	Item #	Checklist item	Location where item is reported
**TITLE**			
Title	1	Identify the report as a systematic review.	First page
**ABSTRACT**			
Abstract	2	See the PRISMA 2020 for Abstracts checklist.	First page
**INTRODUCTION**			
Rationale	3	Describe the rationale for the review in the context of existing knowledge.	"1. Introduction"
Objectives	4	Provide an explicit statement of the objective(s) or question(s) the review addresses.	"1. Introduction"
**METHODS**			
Eligibility criteria	5	Specify the inclusion and exclusion criteria for the review and how studies were grouped for the syntheses.	"2.1. Eligibility criteria", table 1
Information sources	6	Specify all databases, registers, websites, organisations, reference lists and other sources searched or consulted to identify studies. Specify the date when each source was last searched or consulted.	Supplementary table 2-13
Search strategy	7	Present the full search strategies for all databases, registers and websites, including any filters and limits used.	Supplementary table<br>2-9 and 13
Selection process	8	Specify the methods used to decide whether a study met the inclusion criteria of the review, including how many reviewers screened each record and each report retrieved, whether they worked independently, and if applicable, details of automation tools used in the process.	"2.2. Search strategies, information sources and study selection"
Data collection process	9	Specify the methods used to collect data from reports, including how many reviewers collected data from each report, whether they worked independently, any processes for obtaining or confirming data from study investigators, and if applicable, details of automation tools used in the process.	"2.3 Data collection and synthesis of results"
Data items	10a	List and define all outcomes for which data were sought. Specify whether all results that were compatible with each outcome domain in each study were sought (e.g. for all measures, time points, analyses), and if not, the methods used to decide which results to collect.	"2.3 Data collection and synthesis of results" and supplementary table 15
	10b	List and define all other variables for which data were sought (e.g. participant and intervention characteristics, funding sources). Describe any assumptions made about any missing or unclear information.	"2.3 Data collection and synthesis of results" and supplementary table 15
Study risk of bias assessment	11	Specify the methods used to assess risk of bias in the included studies, including details of the tool(s) used, how many reviewers assessed each study and whether they worked independently, and if applicable, details of automation tools used in the process.	"2.4. Risk of bias and quality of evidence"
Effect measures	12	Specify for each outcome the effect measure(s) (e.g. risk ratio, mean difference) used in the synthesis or presentation of results.	"2.3 Data collection and synthesis of results"
Synthesis methods	13a	Describe the processes used to decide which studies were eligible for each synthesis (e.g. tabulating the study intervention characteristics and comparing against the planned groups for each synthesis (item #5)).	"2.3 Data collection and synthesis of results"
	13b	Describe any methods required to prepare the data for presentation or synthesis, such as handling of missing summary statistics, or data conversions.	Not applicable

Section and Topic	Item #	Checklist item	Location where item is reported
	13c	Describe any methods used to tabulate or visually display results of individual studies and syntheses.	Not applicable
	13d	Describe any methods used to synthesize results and provide a rationale for the choice(s). If meta-analysis was performed, describe the model(s), method(s) to identify the presence and extent of statistical heterogeneity, and software package(s) used.	"2.3 Data collection and synthesis of results"
	13e	Describe any methods used to explore possible causes of heterogeneity among study results (e.g. subgroup analysis, meta-regression).	Not applicable
	13f	Describe any sensitivity analyses conducted to assess robustness of the synthesized results.	Not applicable
Reporting bias assessment	14	Describe any methods used to assess risk of bias due to missing results in a synthesis (arising from reporting biases).	Not applicable
Certainty assessment	15	Describe any methods used to assess certainty (or confidence) in the body of evidence for an outcome.	"2.4. Risk of bias and quality of evidence"
**RESULTS**			
Study selection	16a	Describe the results of the search and selection process, from the number of records identified in the search to the number of studies included in the review, ideally using a flow diagram.	"3.1. Selection of studies", figure 1
	16b	Cite studies that might appear to meet the inclusion criteria, but which were excluded, and explain why they were excluded.	Supplementary table 14
Study characteristics	17	Cite each included study and present its characteristics.	"3.2. Study characteristics", table 2-3 and "3.2.1. Intervention details"
Risk of bias in studies	18	Present assessments of risk of bias for each included study.	"3.5. Risk of bias", figure 2-5
Results of individual studies	19	For all outcomes, present, for each study: (a) summary statistics for each group (where appropriate) and (b) an effect estimate and its precision (e.g. confidence/credible interval), ideally using structured tables or plots.	Not applicable
Results of syntheses	20a	For each synthesis, briefly summarise the characteristics and risk of bias among contributing studies.	Not reported
	20b	Present results of all statistical syntheses conducted. If meta-analysis was done, present for each the summary estimate and its precision (e.g. confidence/credible interval) and measures of statistical heterogeneity. If comparing groups, describe the direction of the effect.	Not applicable
	20c	Present results of all investigations of possible causes of heterogeneity among study results.	Not applicable
	20d	Present results of all sensitivity analyses conducted to assess the robustness of the synthesized results.	Not applicable
Reporting biases	21	Present assessments of risk of bias due to missing results (arising from reporting biases) for each synthesis assessed.	Not applicable
Certainty of evidence	22	Present assessments of certainty (or confidence) in the body of evidence for each outcome assessed.	"3.6. Certainty of evidence" and supplementary table 16-38
**DISCUSSION**			
Discussion	23a	Provide a general interpretation of the results in the context of other evidence.	"4. Discussion"
	23b	Discuss any limitations of the evidence included in the review.	"4. Discussion"
	23c	Discuss any limitations of the review processes used.	"4. Discussion"
	23d	Discuss implications of the results for practice, policy, and future research.	Implications for practice and policy: "4- Discussion" Implications for

Section and Topic	Item #	Checklist item	Location where item is reported
			future research: “4. Discussion” and supplementary table 40
**OTHER INFORMATION**			
Registration and protocol	24a	Provide registration information for the review, including register name and registration number, or state that the review was not registered.	“11. Registration”
	24b	Indicate where the review protocol can be accessed, or state that a protocol was not prepared.	“11. Registration”
	24c	Describe and explain any amendments to information provided at registration or in the protocol.	Not applicable
Support	25	Describe sources of financial or non-financial support for the review, and the role of the funders or sponsors in the review.	“7. Acknowledgements” and “10. Funding”
Competing interests	26	Declare any competing interests of review authors.	“8. Conflict of interest”
Availability of data, code and other materials	27	Report which of the following are publicly available and where they can be found: template data collection forms; data extracted from included studies; data used for all analyses; analytic code; any other materials used in the review.	“Data Availability Statement”

2. Search strategies

2.1. Development of search strategies

Supplementary table 2: Development of search strategies

Background sources	The search strategy was developed in accordance with chapter 4 in Cochrane's method book (2) and chapter 4 in the method book by NIPH (3). Librarians at the medical library at University of Bergen were consulted throughout the process.
Selection of search terms	A selection of terms used in a related SR (4), who had adapted the search strategy from a SR on AAT for trauma (5), formed a basis for the search string. IAHAIOs definition of animal-assisted interventions (6) was used for both expanding and delimitation of the strategy. Relevant articles detected through initial, non-systematic searches in Google Scholar and PubMed were reviewed for additional search terms and used for validation of the search strategy (7-14). The search string consisted of both text words and subject words, as well as truncations, proximity operators and Boolean operators.
Selection of the databases	Databases were selected through review of available candidate databases (15, 16) and by consultation of librarians at the medical library at University of Bergen. The selection corresponded with requirements and recommendations in chapter 4 in the handbook by Cochrane (2) and in the handbook by NIPH (3).
Conducting of the search	Searches in titles, abstracts and key words, which is the recommended combination (17), were made for databases where this combination was available. For the remaining databases, searches were conducted in titles and abstracts. No search filters, such as limitations regarding article types, species, language, or age, were used. The main search was conducted 21.05.22 in APA PsycInfo (Ovid), AMED (Ovid), CENTRAL (Cochrane), Cinahl (Ebsco), Embase (Ovid), Medline (Ovid) and Web of Science. Automatic alerts regarding novel publications until submission of the manuscript were set up. Supplementary searches were conducted in CAB abstracts, ClinicalTrials.gov, DANS (Data Archiving and Networked Services), HABRI central, NORA (Norwegian Open Research Archives), OAIster, and ProQuest. Websites of Affinity foundation (18), IAHIO (19), and Therapy Animals vzw (20) were also searched for both published and grey literature, in addition to updated searches in Google Scholar.

Validation	PRESS (Peer Review Of Electronic Search Strategies) 2015 guidelines were used for final validation of the strategy (21). In addition, librarians at the at medical library at University of Bergen were consulted.

2.2. Main searches

The main searches were conducted 21.05.2022

Supplementary table 3: Ovid MEDLINE(R) and Epub Ahead of Print, In-Process, In-Data-Review & Other Non-Indexed Citations and Daily <1946 to May 20, 2022>

1	exp schizophrenia/	112067
2	schizophrenic psychology/	34258
3	psychotic disorders/	50305
4	schizo*.ti,ab,kf.	156423
5	psychos?s.ti,ab,kf.	55340
6	((psychotic or delusional) adj disorder*).ti,ab,kf.	11645
7	((positive or negative) adj symptom*).ti,ab,kf.	14216
8	(severe adj (mental or psychiatric)).ti,ab,kf.	11829
9	animal assisted therapy/	516
10	exp dog/	338173
11	exp human-animal bond/	1983
12	((animal* or pet or pets or dog or dogs or canine* or canis or canid* or puppy or puppies) adj3 (therap* or activit* or coach* or assist* or interaction* or intervention* or facilitat* or visit* or resident*)).ti,ab,kf.	30342
13	((psychiatric or mental or emotional) adj3 (animal* or pet or pets or dog or dogs or canine* or canis or canid* or puppy or puppies)).ti,ab,kf.	1073
14	(human adj2 (animal* or pet or pets or dog or dogs or canine* or canis or canid* or puppy or puppies) adj2 bond*).ti,ab,kf.	324
15	((animal* or pet or pets or dog or dogs or canine* or canis or canid* or puppy or puppies) and (aai or aat or aaa)).ti,ab,kf.	1306
16	biophilia hypothes*.ti,ab,kf.	21
17	1 or 2 or 3 or 4 or 5 or 6 or 7 or 8	235747
18	9 or 10 or 11 or 12 or 13 or 14 or 15 or 16	364653
19	17 and 18	619

Supplementary table 4: Embase <1974 to 2022 May 20>

1	exp schizophrenia spectrum disorder/	197276
2	psychosis/	96171
3	acute psychosis/	2006
4	paranoid psychosis/	1718
5	schizo*.ti,ab,kf.	200162
6	psychos?s.ti,ab,kf.	72068
7	((psychotic or delusional) adj disorder*).ti,ab,kf.	18613
8	((positive or negative) adj symptom*).ti,ab,kf.	21882
9	(severe adj (mental or psychiatric)).ti,ab,kf.	16016
10	animal assisted therapy/	708
11	exp pet therapy/	242
12	exp dog/	279293
13	exp human-animal bond/	625
14	((animal* or pet or pets or dog or dogs or canine* or canis or canid* or puppy or puppies) adj3 (therap* or activit* or coach* or assist* or interaction* or intervention* or facilitat* or visit* or resident*)).ti,ab,kf.	39130
15	((psychiatric or mental or emotional) adj3 (animal* or pet or pets or dog or dogs or canine* or canis or canid* or puppy or puppies)).ti,ab,kf.	1263
16	(human adj2 (animal* or pet or pets or dog or dogs or canine* or canis or canid* or puppy or puppies) adj2 bond*).ti,ab,kf.	364
17	((animal* or pet or pets or dog or dogs or canine* or canis or canid* or puppy or puppies) and (aai or aat or aaa)).ti,ab,kf.	1901
18	biophilia hypothes*.ti,ab,kf.	18
19	1 or 2 or 3 or 4 or 5 or 6 or 7 or 8 or 9	326781
20	10 or 11 or 12 or 13 or 14 or 15 or 16 or 17 or 18	313744
21	19 and 20	844

Supplementary table 5: APA PsycInfo <1806 to May Week 3 2022>

1	exp schizophrenia/	95896
2	psychosis/	30770
3	acute psychosis/	1306
4	chronic psychosis/	244
5	"paranoia (psychosis)"/	1292
6	schizo*.ti,ab,id.	135926
7	psychos?s.ti,ab,id.	57875
8	((psychotic or delusional) adj disorder*).ti,ab,id.	11799
9	((positive or negative) adj symptom*).ti,ab,id.	13261
10	(severe adj (mental or psychiatric)).ti,ab,id.	11213
11	animal assisted therapy/	1193
12	pets/	1886
13	exp dogs/	5758
14	interspecies interaction/	4792
15	((animal* or pet or pets or dog or dogs or canine* or canis or canid* or puppy or puppies) adj3 (therap* or activit* or coach* or assist* or interaction* or intervention* or facilitat* or visit* or resident*)).ti,ab,id.	6299
16	((psychiatric or mental or emotional) adj3 (animal* or pet or pets or dog or dogs or canine* or canis or canid* or puppy or puppies)).ti,ab,id.	1180
17	(human adj2 (animal* or pet or pets or dog or dogs or canine* or canis or canid* or puppy or puppies) adj2 bond*).ti,ab,id.	391
18	((animal* or pet or pets or dog or dogs or canine* or canis or canid* or puppy or puppies) and (aai or aat or aaa)).ti,ab,id.	440
19	biophilia hypothes*.ti,ab,id.	43
20	1 or 2 or 3 or 4 or 5 or 6 or 7 or 8 or 9 or 10	185233
21	11 or 12 or 13 or 14 or 15 or 16 or 17 or 18 or 19	15576
22	20 and 21	290

Supplementary table 6: AMED (Allied and Complementary Medicine) <1985 to May 2022>

1	exp schizophrenia/	1010
2	psychotic disorders/	463
3	schizo*.ti,ab.	1231
4	psychos?s.ti,ab.	355
5	((psychotic or delusional) adj disorder*).ti,ab.	109
6	((positive or negative) adj symptom*).ti,ab.	112
7	(severe adj (mental or psychiatric)).ti,ab.	557
8	animal assisted therapy/	101
9	dogs/	313
10	bonding human pet/	48
11	((animal* or pet or pets or dog or dogs or canine* or canis or canid* or puppy or puppies) adj3 (therap* or activit* or coach* or assist* or interaction* or intervention* or facilitat* or visit* or resident*)).ti,ab.	385
12	((psychiatric or mental or emotional) adj3 (animal* or pet or pets or dog or dogs or canine* or canis or canid* or puppy or puppies)).ti,ab.	23
13	(human adj2 (animal* or pet or pets or dog or dogs or canine* or canis or canid* or puppy or puppies) adj2 bond*).ti,ab.	16
14	((animal* or pet or pets or dog or dogs or canine* or canis or canid* or puppy or puppies) and (aai or aat or aaa)).ti,ab.	20
15	biophilia hypothes*.ti,ab.	1
16	1 or 2 or 3 or 4 or 5 or 6 or 7	2395
17	8 or 9 or 10 or 11 or 12 or 13 or 14 or 15	712
18	16 and 17	5

Supplementary table 7: Web of Science

13	#11 AND #12	422
12	#12 #6 OR #7 OR #8 OR #9 OR #10	36,729
11	#11 #1 OR #2 OR #3 OR #4 OR #5	287,034
10	TS=(biophilia hypothes*)	93
9	TS=((animal* or pet or pets or dog or dogs or canine* or canis or canid* or puppy or puppies) and (aai or aat or aaa))	1,497
8	#8 TS=((human NEAR/1 (animal* or pet or pets or dog or dogs or canine* or canis or canid* or puppy or puppies) NEAR/1 bond*))	558
7	TS=((psychiatric or mental or emotional) NEAR/2 (animal* or pet or pets or dog or dogs or canine* or canis or canid* or puppy or puppies))	1,338
6	TS=((animal* or pet or pets or dog or dogs or canine* or canis or canid* or puppy or puppies) NEAR/2 (therap* or activit* or coach* or assist* or interaction* or intervention* or facilitat* or visit* or resident*))	34,004
5	TS=(severe NEAR/0 (mental or psychiatric))	14,551
4	TS=((positive or negative) NEAR/0 symptom*)	18,832
3	TS=((psychotic or delusional) NEAR/0 disorder*)	13,390

2	TS=(psychos$s)	68,934
1	TS=(schizo*)	239,528

Supplementary table 8: Cinahl

33	S31 AND S32	75
32	S15 OR S16 OR S17 OR S18 OR S19 OR S20 OR S21 OR S22 OR S23 OR S24 OR S25 OR S26 OR S27 OR S28 OR S29 OR S30	16,920
31	S1 OR S2 OR S3 OR S4 OR S5 OR S6 OR S7 OR S8 OR S9 OR S10 OR S11 OR S12 OR S13 OR S14	55,813
30	AB "biophilia hypothes*"	5
29	TI "biophilia hypothes*"	0
28	AB ((animal* or pet or pets or dog or dogs or canine* or canis or canid* or puppy or puppies) and (aai or aat or aaa))	289
27	TI ((animal* or pet or pets or dog or dogs or canine* or canis or canid* or puppy or puppies) and (aai or aat or aaa))	13
26	AB ((human N1 (animal* or pet or pets or dog or dogs or canine* or canis or canid* or puppy or puppies) N1 bond*))	84
25	TI ((human N1 (animal* or pet or pets or dog or dogs or canine* or canis or canid* or puppy or puppies) N1 bond*))	50
24	AB ((psychiatric or mental or emotional) N2 (animal* or pet or pets or dog or dogs or canine* or canis or canid* or puppy or puppies))	166
23	TI ((psychiatric or mental or emotional) N2 (animal* or pet or pets or dog or dogs or canine* or canis or canid* or puppy or puppies))	65
22	AB ((animal* or pet or pets or dog or dogs or canine* or canis or canid* or puppy or puppies) N2 (therap* or activit* or coach* or assist* or interaction* or intervention* or facilitat* or visit* or resident*))	3,076
21	TI ((animal* or pet or pets or dog or dogs or canine* or canis or canid* or puppy or puppies) N2 (therap* or activit* or coach* or assist* or interaction* or intervention* or facilitat* or visit* or resident*))	1,214
20	MH human-pet bonding	965
19	MH dogs	10,778
18	MH pets	2,959
17	MH pet therapy	1,493
16	MH therapy animals	20
15	MH "Animal Assisted Therapy (Iowa NIC)"	1
14	AB (severe N0 (mental or psychiatric))	4,194
13	TI (severe N0 (mental or psychiatric))	2,010
12	AB ((positive or negative) N0 symptom*)	3,879
11	TI ((positive or negative) N0 symptom*)	704
10	AB ((psychotic or delusional) N0 disorder*)	3,518
9	TI ((psychotic or delusional) N0 disorder*)	979
8	AB psychos?s	11,654
7	TI psychos?s	8,100
6	AB schizo*	24,482
5	TI schizo*	20,415
4	MH schizoaffective disorder	379
3	MH paranoid disorders	808
2	MH psychotic disorders	14,077
1	MH schizophrenia	27,752

Supplementary table 9: Cochrane (CENTRAL)

1	MeSH descriptor: [Schizophrenia] explode all trees	7993
2	MeSH descriptor: [Schizophrenia Spectrum and Other Psychotic Disorders] this term only	28
3	schizo*:ti,ab,kw	19092
4	psychos?s:ti,ab,kw	7079
5	((psychotic or delusional) NEXT disorder*):ti,ab,kw	4248
6	(positive or negative) NEXT symptom*:ti,ab,kw	3637
7	(severe NEXT (mental or psychiatric)):ti,ab,kw	1629
8	MeSH descriptor: [Animal Assisted Therapy] this term only	44
9	MeSH descriptor: [Therapy Animals] explode all trees	4
10	MeSH descriptor: [Dogs] this term only	508
11	((animal* or pet or pets or dog or dogs or canine* or canis or canid* or puppy or puppies) NEAR/3 (therap* or activit* or coach* or assist* or interaction* or intervention* or facilitated or visit* or resident*)):ti,ab,kw	2082
12	((psychiatric or mental or emotional) NEAR/3 (animal* or pet or pets or dog or dogs or canine* or canis or canid* or puppy or puppies)):ti,ab,kw	46
13	(human NEAR/2 (animal* or pet or pets or dog or dogs or canine* or canis or canid* or puppy or puppies) NEAR/2 bond*):ti,ab,kw	38
14	((animal* or pet or pets or dog or dogs or canine* or canis or canid* or puppy or puppies) and (aai or aat or aaa)):ti,ab,kw	124
15	(biophilia hypothes*):ti,ab,kw	2
16	#1 or #2 or #3 or #4 or #5 or #6 or #7	24832
17	#8 or #9 or #10 or #11 or #12 or #13 or #14 or #15	2589
18	#16 and #17	41

2.3. Supplementary searches

Supplementary table 10: Forward citation searching via Google Scholar

Article	Date for most updated search	Results
Barker and Dawson, 1998, (22)	14.05.22	719
Calvo et al., 2016 (7)	07.05.22	58
Chen et al., 2021 (12)	06.05.22	1
Chen et al., 2022 (23)	25.06.22	0
Chu et al., 2009 (14)	14.05.22	122
Kovacs et al., 2004 (8)	04.05.22	170
Kovacs et al., 2006 (9)	03.05.22	54
Lang et al., 2010 (10)	14.05.22	92
Monfort et al., 2022 (24)	25.06.22	0
Nathans-Barel et al., 2005 (11)	06.05.22	201
Shih et al., 2023 (25)	19.03.23	0
Villalta-Gil et al., 2009 (13)	30.04.22	81

Supplementary table 11: Backward citation searches in the included articles

Article	Results
Barker and Dawson, 1998 (22)	31
Calvo et al., 2016 (7)	59
Chen et al., 2021 (12)	65
Chen et al., 2022 (23)	45
Chu et al., 2009 (14)	38
Kovacs et al., 2004 (8)	15
Kovacs et al., 2006 (9)	32
Lang et al., 2010 (10)	29
Monfort et al., 2022 (24)	44
Nathans-Barel et al., 2005 (11)	37
Shih et al., 2023, (25)	32
Villalta-Gil et al., 2009 (13)	43

Supplementary table 12: Backward citation searches in some relevant reviews detected by the main search

Article	Results
Barker and Wolen, 2008 (26)	110
Bernabei et al., 2013 (27)	86
Cakici and Kok, 2020 (28)	60
Charry-Sanchez et al., 2018: (29)	66
Cirulli et al., 2011: (30)	108
Cherniack and Cherniack, 2014 (31)	65
Dimitrijevic, 2009 (32)	24
Hawkins et al., 2019 (33)	69
Kamioka et al., 2014 (34)	36
Koukourikos et al., 2019 (35)	42
Lasa et al., 2011 (36)	50
Maujean et al., 2015 (37)	31
Peluso et al., 2018 (38)	38
Rossetti and King, 2010, (39)	19
Spattini et al., 2018 (40)	34
Virues-Ortega et al., 2012 (41)	72

Supplementary table 13: Searches in websites of organizations and other databases

Organization/database	Search terms/method	Date for most updated search	Results
Affinity foundation (18)	Review of all cited publications	18.06.22	7
CAB abstracts	"schizophrenia" or "schizoaffective" or "schizotypal" or "delusional" or "psychosis" or "psychoses" or "psychotic"	19.06.22	0
ClinicalTrials.gov	Combinations of terms in the fields “condition of disease” and “other terms”<br>- Schizophrenia and animal-assisted<br>- Schizophrenia and dog<br>Schizophrenia and canine	18.06.22	3

	- Psychosis; Schizophrenia-Like and animal-assisted<br>- Psychosis; Schizophrenia-Like and dog<br>- Psychosis; Schizophrenia-Like and canine		
DANS (Data Archiving and Networked Services)	("schizophrenia" OR "schizoaffective" OR "schizotypal" OR "delusional" OR "psychosis" OR "psychoses" OR "psychotic") AND ("animal-assisted" OR "dog-assisted" OR "canine-assisted" OR "therap* animal" OR "therap* dog")	19.06.22	0
Google Scholar	("schizophrenia" OR "schizoaffective" OR "schizotypal" OR "delusional" OR "psychosis" OR "psychoses" OR "psychotic") AND ("animal-assisted" OR "dog-assisted" OR "canine-assisted" OR "therap* animal" OR "therap* dog"))	28.05.23	Sorted by relevance, review of the first 50 articles
HABRI central	"schizophrenia" "schizoaffective" "schizotypal" "delusional" "psychosis" "psychoses" "psychotic"	18.06.22	7
IAHAIO (19)	Review of all research articles (vol.1 – vol-5)	18.06.22	22
NORA (Norwegian Open Research Archives)	"animal-assisted" or "dyreassistert*" or "dog-assisted" or "canine-assisted" or "hundeassistert*" or "therap* animal*" or "terapidyr" or "therap* dog*" or "therap* canine" or "terapihund*"	19.06.22	29
OAIster	"animal-assisted"<br>"dog-assisted"<br>"canine-assisted"<br>"therap* animal"<br>"therap* dog"	19.06.22	3
ProQuest	Search filter: exclusion of books<br><br>(AB,TI ("schizophrenia" OR "schizoaffective" OR "schizotypal" OR "delusional" OR "psychosis" OR "psychoses" OR "psychotic")) AND (AB,TI ("animal-assisted" OR "dog-assisted" OR "canine-assisted" OR "therap* animal" OR "therap* dog"))	19.06.22	35
Therapy Animals vzw (20)	Review of "reading material"	25.06.22	257

3. Excluded articles

Supplementary table 14: Excluded after review in full-text version or due to lack of full-text version

Articles	Reason for exclusion	Some sources
Barak et al., 2001: «Animal assisted therapy for elderly schizophrenic patients: A one-year controlled trial» (42)	Lack of distinguishing between measurements from interventions with dogs and cats	Systematic searches in Cochrane (CENTRAL), Embase, Medline, PsycINFO and Web of science
Barker et al., 2003: "Effects of Animal-Assisted Therapy on Patients' Anxiety, Fear, and Depression Before ECT" (43)	Lack of distinguishing between measurements from participants diagnosed with psychotic disorders, depression, bipolar disorders and dementia with depression	Review of references (10, 22, 36, 39)
Barnes, 1986: «The effects of pet facilitated therapy on social self care behaviors of schizophrenics» (44)	No access to full-text version, animal specie not specified in abstract	Systematic search in PsycINFO
Bauman, 1991: "The effects of animal-assisted therapy on communication patterns with chronic schizophrenics" (45)	Did not include dogs, lack of distinguishing between measurements from participants diagnosed with schizophrenia and other disorders	Review of "reading material" (20)
Brown et al., 2019: "Effects of Animal-Assisted Activity on Mood States and Feelings in a Psychiatric Setting" (46)	Lack of distinguishing between measurements from participants diagnosed with depression, bipolar disorder, anxiety disorder and psychoses	Forward citation searching (7, 11, 13)
Chang et al., 2015: "The healing process of chronic schizophrenic patients in animal-assisted therapy" (47)	No access to full-text version	Review of references (25)
Corson et al., 1977: "Pet dogs as nonverbal communication links in hospital psychiatry" (48)	Overall critical risk of bias (critical risk regarding confounding, low risk regarding selection of participants, low risk regarding classification of interventions, no information regarding deviations from intended interventions, serious risk regarding missing data, critical risk regarding measurements of outcomes, and serious risk regarding selection of the reported results). The risk of bias was assessed through ROBINS-I (49).	Review of "reading material" (20), and review of references (39)

Cox, 1999: "Pet-facilitated occupational therapy: Efficacy in a psychiatric setting" (50)	Lack of distinguishing between measurements from participants diagnosed with psychosis, obsessive-compulsive disorder, schizoaffective disorder, dissociative identity disorder, dementia, chronic pain and eating disorder.	Forward citation searching (22)
Eklund and Johansson, 2014: "Det här är Hilda, hon är Uppsalas bästa tjej"-En fallstudie om vårdhundens roll i socialt arbet" (51)	Not a quantitative study	Forward citation searching (11, 14)
Hall and Malpus, 2000: "Pets as therapy: Effects on social interaction in long-stay psychiatry" (52)	Participants with severe and enduring psychiatric illness, diagnoses not further specified	Review of references (34)
Haughie et al., 1992: "An evaluation of companion pets with elderly psychiatric patients" (53)	Lack of distinguishing between measurements from participants diagnosed with schizophrenia, depression, bipolar disorder and dementia	Systematic search in Embase
Henry and Crowley, 2015: "The Psychological and Physiological Effects of Using a Therapy Dog in Mindfulness Training", (54)	Diagnoses not specified	Forward citation searching (22)
Holcomb and Meacham, 1989: "Effectiveness of an Animal-Assisted Therapy Program in an Inpatient Psychiatric Unit" (55)	No access to full-text version	Review of "reading material" (20), review of references (33, 37)
Hudson, 2016: "Animal assisted therapy and the effects on anxiety and behavioral symptoms for geriatric patients living in a facility" (56)	Lack of distinguishing between measurements from participants diagnosed with schizophrenia, dementia and bipolar disorder	Forward citation searching (10)
Kung et al., 2005: "The evaluation of pet therapy on negative symptoms in inpatients with chronic schizophrenia" (57)	No access to full text version. Included hamsters according to Hawkins et al. (33)	Systematic search in Cochrane (CENTRAL)
LaJoie, 2003: "An evaluation of the effectiveness of using animals in therapy. Thesis dissertation" (58)	No access to full text version	Review of references (40)
Levinson, 1962: "The dog as a "co-therapist" (59)	Children diagnosed with schizophrenia	Review of references (22), review of "reading material" (20)
Marques et al., 2015: "Effectiveness of animal-assisted interventions in preventing violence in acute psychiatric inpatients" (60)	Lack of distinguishing between measurements from participants with psychotic disorders, mood disorders and other disorders	Forward citation searching (10, 14)
Marr et al., 2000: "Animal-Assisted Therapy in Psychiatric Rehabilitation" (61)	Lack of distinguishing between measurements from participants diagnosed with schizophrenia, psychosis NOS, bipolar disorder and depression. Lack of distinguishing between measurements from interventions with dogs, rabbits, ferrets and guinea pigs.	Forward citation searching (22), review of references (26, 30, 33, 34, 37)
Montolio and Sancho-Pelluz, 2020: "Animal-assisted therapy in the residential treatment of dual pathology" (62)	Lack of distinguishing between measurements from participants with schizophrenia spectrum disorders, mood disorders, personality disorders, bipolar disorder and other mental disorders.	Systematic search in Embase
Moretti et al., 2010: "A pet therapy intervention on elderly inpatients: An epidemiological study" (63)	Conference abstract	Systematic search in Embase
Moretti et al., 2011: "Pet therapy in elderly patients with mental illness" (64)	Lack of distinguishing between measurements from participants with psychotic disorders, Alzheimer's disease, vascular dementia, secondary dementia and mood disorder	Systematic search in Embase, Cochrane (CENTRAL), Medline, PsycINFO and Web of science
Morgan, 2008: "An examination of the anxiolytic effects of interaction with a therapy dog" (65)	Did not include participants with schizophrenia and related disorders	Forward citation searching (8, 11, 22)
Mulvaney-Roth et al., 2022: "Using Pet Therapy to Decrease Patients' Anxiety on Two Diverse Inpatient Units" (66)	Diagnoses not specified	Forward citation searching (22)
Nagendrappa et al., 2020: "Recognizing the role of animal-assisted therapies in addressing mental health needs during the COVID-19 pandemic" (67)	Not a quantitative study	Systematic search in Embase
Nepps et al., 2014: «Animal-Assisted Activity: Effects of a Complementary Intervention Program on Psychological and Physiological Variables» (68)	Lack of distinguishing between measurements from participants diagnosed with schizophrenia and affective disorders	Review of references (7, 30), forward citation searching (22)
Nurenberg et al., 2015: "Animal-assisted therapy with chronic psychiatric inpatients: equine-assisted psychotherapy and aggressive behavior" (69)	Lack of distinguishing between measurements from participants diagnosed with schizophrenia, schizoaffective disorder and affective disorders	Systematic search in Cochrane (CENTRAL), Cinahl, Embase, Medline, PsycINFO and Web of science

Pollock et al., 2017: "Animals as icebreakers: A pilot animal-assisted therapy group for veterans with serious mental illness" (70)	Lack of distinguishing between measurements from interventions with different animal species	Forward citation searching (8)
Saludes, 2018: "Canines in Clinical Settings: Assessing the Psychosocial Outcomes of Therapy Dog Inclusion during a Stressful Interview", (71)	Did not include participants with psychotic disorders	Forward citation searching (22)
Stefanini et al., 2015: "The use of Animal-Assisted Therapy in adolescents with acute mental disorders: A randomized controlled study" (72)	Lack of distinguishing between measurements from participants diagnosed with schizophrenia, mood disorders, anxiety disorders and eating disorders	Search in Google Scholar, review of references (33)
Thompson et al., 1983: "Pets as socializing agents with chronic psychiatric patients: An initial study" (73)	No access to full-text version	Review of "reading material" (20)
Tribulato, 2004: "Animal -assisted therapy for uncommunicative psychiatric patients with catatonic features" (74)	Not a quantitative study	Forward citation searching (22)
Venkatesan, 2019: "Pawsonalised medicine: animals for mental health" (75)	No access to full-text version	Systematic search in Embase
Villalta-Gil et al., 2006: "Pet-assisted therapy for inpatients with diagnosis of schizophrenia" (76)	Meeting abstract of a literature review	Systematic search in Web of science
Zisselman et al., 1996: "A Pet Therapy Intervention With Geriatric Psychiatry Inpatients" (77)	Lack of distinguishing between measurements from participants diagnosed with schizophrenia, major depression, delirium, primary degenerative dementia with delusions, multi-infarct dementia with depression, primary degenerative dementia with depression, multi-infarct dementia with delusions, adjustment disorders, bipolar disorder and psychosis not otherwise specified	Review of references (26, 31, 33, 34, 38)

4. Data elements

Supplementary table 15: Data elements

**Population**	- Diagnosis, diagnostic system, acute or chronic<br>- Age, gender, duration of illness, baseline treatment, treatment situation (hospitalization, outpatient clinic or social institution)<br>- Number of participants included in the study (intervention group and control group), number of participants included in the analyses (intervention group and control group)
**Intervention**	Described on the basis of TIDieR (Template for Intervention Description and Replication) (78) and modified:<br>- Description of the intervention<br>- Aim<br>- Description of the dogs: education/experience, approval/certification<br>- Key elements of the intervention<br>- Duration and frequency, number of sessions<br>- Personnel: Education/experience<br>- Group size<br>- Localization: Country, hospital/outpatient clinic/social institution, indoors/outdoors
**Comparison**	- Description of program<br>- Duration and frequency, number of sessions
**Outcomes**	- Outcomes related to effect of intervention: Change in symptoms, such as positive, negative and cognitive, and related aspects such as somatic health, stress, quality of life and functioning in everyday life
**Other properties**	- Year of publication<br>- Study design

5. Quality assessments

The assessments were based on recommendations from GRADE ("Grading of Recommendations Assessment, Development and Evaluation") handbook (79), an article

regarding imprecision (80) and an article regarding rating of quality of evidence in SRs without metanalysis (81).

5.1. General symptomatology

Measured with PANSS (7, 12, 13, 24). Limitations related to consistency, precision and risk of bias led to downgrading. The quality of evidence was considered **very low.**

Supplementary table 16: General symptomatology

	Limitations	Assessment
**Consistency**	Serious limitations	One study showed significant improvement between the intervention group and the control group (12). One study showed significant improvement within both groups, but not between the groups (7). One study showed significant improvement only within the control group (13). One study showed no significant improvement between the groups (24).<br><br>It cannot be ruled out that heterogeneity regarding study characteristics can explain part of the inconsistency.
**Directness**	No serious limitations	Relevant study characteristics (population, interventions, comparison and outcome)
**Precision**	Serious limitations	Data from four studies, less than 800 participants
**Publication bias**	Not suspected	The material included both studies with significant findings and studies with non-significant findings. No findings of studies reported in trial registers not published.
**Limitations in study design or execution**	Very serious limitations	High risk of bias in three of the studies, serious risk of bias in one

5.2. Positive symptoms

Measured with PANSS (7, 11-13, 24) and with a questionnaire (14). Limitations related to consistency, precision and risk of bias led to downgrading. The quality of evidence was considered **very low.**

Supplementary table 17: Positive symptoms

	Limitations	Assessment
**Consistency**	Serious limitations	Three studies showed significant improvement for the intervention group compared with the control group (10, 12, 24). SRD was 0.15 in one of the studies, indicating small effect size (12). Two of the studies showed significant improvement both within the intervention group and within the control group, and no significant difference was found between the groups (7, 13). Cohen's *d* was 1.08 in one of the studies for both groups, indicating large effect size (13). One study found no significant difference between the groups for positive symptoms, and significance within the groups was not stated (11).<br><br>It cannot be ruled out that heterogeneity regarding study characteristics can explain part of the inconsistency.
**Directness**	No serious limitations	Relevant study characteristics (population, interventions, comparison and outcomes)
**Precision**	Serious limitations	Data from six studies, less than 800 participants
**Publication bias**	Not suspected	The material included both studies with significant findings and studies with non-significant findings. No findings of studies reported in trial registers not published.
**Limitations in study design or execution**	Very serious limitations	High risk of bias in four of the studies, serious risk of bias in two

5.3. Negative symptoms

Measured with PANSS (7, 12, 13, 24), with a questionnaire (14), and with SANS (11). Limitations related to consistency, precision and risk of bias led to downgrading. The quality of evidence was considered **very low.**

Supplementary table 18: Negative symptoms

	Limitations	Assessment
**Consistency**	Serious limitations	One study showed significant improvement, with SRD 0.50 indicating large effect size, for the intervention group compared with the control group (12). Two studies showed significant improvement within the intervention groups, and not within the control groups. (7, 13). Cohen's *d* was 1.64 in one of the studies, indicating large effect size (13). The difference between the groups in the two studies was not significant. Three studies found no significant change between the groups for negative symptoms, and significance within the groups was not stated (10, 11, 24) .<br><br>It cannot be ruled out that heterogeneity regarding study characteristics can explain part of the inconsistency.
**Directness**	No serious limitations	Relevant study characteristics (population, interventions, comparison and outcomes)
**Precision**	Serious limitations	Data from six studies, less than 800 participants
**Publication bias**	Not suspected	The material included both studies with significant findings and studies with non-significant findings. No findings of studies reported in trial registers not published.
**Limitations in study design or execution**	Very serious limitations	High risk of bias in four of the studies, serious risk of bias in two

5.4. Anhedonia

Measured with SHAPS (11). Limitations related to precision and risk of bias led to downgrading. The quality of evidence was considered **very low**.

Supplementary table 19: Anhedonia

	Limitations	Assessment
**Consistency**	N/A	Not possible to assess as the data only came from one study
**Directness**	No serious limitations	Relevant study characteristics (population, intervention, comparison and outcome)
**Precision**	Very serious limitations	Data from one study, less than 800 participants
**Publication bias**	Not suspected	No findings of studies reported in trial registers not published.
**Limitations in study design or execution**	Very serious limitations	Serious risk of bias

5.5. Emotional symptoms

Measured with a questionnaire (14). Limitations related to precision and risk of bias led to downgrading. The quality of evidence was considered **very low**.

Supplementary table 20: Emotional symptoms

	Limitations	Assessment
**Consistency**	N/A	Not possible to assess as the data only came from one study
**Directness**	No serious limitations	Relevant study characteristics (population, intervention, comparison and outcome)
**Precision**	Very serious limitations	Data from one study, less than 800 participants
**Publication bias**	Not suspected	No findings of studies reported in trial registers not published

**Limitations in study design or execution**	Very serious limitations	High risk of bias

5.6. Depression

Measured with DASS-21 (12). Limitations related to precision and risk of bias led to downgrading. The quality of evidence was considered **very low**.

Supplementary table 21: Depression

	**Limitations**	**Assessment**
**Consistency**	N/A	Not possible to assess as the data only came from one study
**Directness**	No serious limitations	Relevant study characteristics (population, intervention, comparison and outcome)
**Precision**	Very serious limitations	Data from one study, less than 800 participants
**Publication bias**	Not suspected	No findings of studies reported in trial registers not published
**Limitations in study design or execution**	Very serious limitations	High risk of bias

5.7. Anxiety

Measured with STAI (10, 22), and with DASS-21 (12). Limitations related to consistency, precision and risk of bias led to downgrading. The quality of evidence was considered **very low**.

Supplementary table 22: Anxiety

	**Limitations**	**Assessment**
**Consistency**	Serious limitations	One study showed significant improvement for the intervention group compared with the control group (10). One study showed significant improvement within the intervention group, and not within the control group, but there was no significant difference between the groups (22). One study found no significant improvement, and significance within the groups was not stated (12).<br><br>It cannot be ruled out that heterogeneity regarding study characteristics can explain part of the inconsistency.
**Directness**	No serious limitations	Relevant study characteristics (population, interventions, comparison and outcome)
**Precision**	Serious limitations	Data from three studies, less than 800 participants
**Publication bias**	Not suspected	The material included both studies with significant findings and studies with non-significant findings. No findings of studies reported in trial registers not published.
**Limitations in study design or execution**	Very serious limitations	Serious risk of bias

5.8. Stress

Measured with DASS-21 (12), and with measurements of cortisol and alpha-amylase (7). Limitations related to directness, precision and risk of bias led to downgrading. The quality of evidence was considered **very low**.

Supplementary table 23: Stress

	**Limitations**	**Assessment**
**Consistency**	No serious limitations	One study showed significant improvement, with SRD 0.15 indicating small effect size, for the intervention group compared with the control group (12). One study showed significant improvement within the intervention group for

		change in cortisol levels. No significance was found for change in alpha-amylase. Levels of cortisol and alpha-amylase were not investigated within the control group (7).
**Directness**	Serious limitations	Cortisol and alpha-amylase: Surrogate outcomes
**Precision**	Serious limitations	Data from two studies, less than 800 participants
**Publication bias**	Not suspected	The material included both studies with significant findings and studies with non-significant findings. No findings of studies reported in trial registers not published.
**Limitations in study design or execution**	Very serious limitations	High risk of bias

5.9. Non-verbal communication

Measured with BGRS (9). Limitations related to precision and risk of bias led to downgrading. The quality of evidence was considered **very low**.

Supplementary table 24: Non-verbal communication

**Element**	**Limitations**	**Assessment**
**Consistency**	N/A	Not possible to assess as the data only came from one study
**Directness**	No serious limitations	Relevant study characteristics (population, intervention and outcome)
**Precision**	Very serious limitations	Data from one study, less than 800 participants
**Publication bias**	Not suspected	No findings of studies reported in trial registers not published
**Limitations in study design or execution (risk of bias)**	Very serious limitations	Serious risk of bias

5.10. Self-esteem

Measured with a questionnaire (14). Limitations related to precision and risk of bias led to downgrading. The quality of evidence was considered **very low**.

Supplementary table 25: Self-esteem

	**Limitations**	**Assessment**
**Consistency**	N/A	Not possible to assess as the data only came from one study
**Directness**	No serious limitations	Relevant study characteristics (population, intervention, comparison and outcome)
**Precision**	Very serious limitations	Data from one study, less than 800 participants
**Publication bias**	Not suspected	No findings of studies reported in trial registers not published
**Limitations in study design or execution**	Very serious limitations	High risk of bias

5.11. Self-determination

Measured with a questionnaire (14). Limitations related to precision and risk of bias led to downgrading. The quality of evidence was considered **very low.**

Supplementary table 26: Self-determination

	**Limitations**	**Assessment**
**Consistency**	N/A	Not possible to assess as the data only came from one study
**Directness**	No serious limitations	Relevant study characteristics (population, intervention, comparison and outcome)
**Precision**	Very serious limitations	Data from one study, less than 800 participants
**Publication bias**	Not suspected	No findings of studies reported in trial registers not published

**Limitations in study design or execution**	Very serious limitations	High risk of bias

5.12. Living skills

Measured with LSP (13), with LSP-20 (24) and with ILSS (8). Limitations related to consistency, precision and risk of bias led to downgrading. The quality of evidence was considered **very low**.

Supplementary table 27: Living skills

	Limitations	Assessment
**Consistency**	Serious limitations	One study showed significant improvement for intervention group compared with control group (24). No significant improvement between the groups was found in another study(13). One study showed significant improvement within the intervention group regarding few of the domains (8).<br><br>It cannot be ruled out that heterogeneity regarding study characteristics can explain part of the inconsistency.
**Directness**	No serious limitations	Relevant study characteristics (population, intervention, comparison and outcome)
**Precision**	Serious limitations	Data from three studies, less than 800 participants
**Publication bias**	Not suspected	No findings of studies reported in trial registers not published
**Limitations in study design or execution**	Very serious limitations	High risk of bias in one of the studies, serious risk of bias in two

5.13. Social function

Measured with MHSFS (25). Limitations related to precision and risk of bias led to downgrading. The quality of evidence was considered **very low.**

Supplementary table 28: Social function

	Limitations	Assessment
**Consistency**	N/A	Not possible to assess as the data only came from one study
**Directness**	No serious limitations	Relevant study characteristics (population, intervention, comparison and outcome)
**Precision**	Very serious limitations	Data from one study, less than 800 participants
**Publication bias**	Not suspected	No findings of studies reported in trial registers not published
**Limitations in study design or execution**	Serious limitations	Some concerns regarding risk of bias

5.14. Social adaptive function

Measured with SAFS (25). Limitations related to precision and risk of bias led to downgrading. The quality of evidence was considered **very low.**

Supplementary table 29: Social adaptive function

	Limitations	Assessment
**Consistency**	N/A	Not possible to assess as the data only came from one study
**Directness**	No serious limitations	Relevant study characteristics (population, intervention, comparison and outcome)
**Precision**	Very serious limitations	Data from one study, less than 800 participants
**Publication bias**	Not suspected	No findings of studies reported in trial registers not published

**Limitations in study design or execution**	Serious limitations	Some concerns regarding risk of bias

5.15. Communication and interaction skills

Measured with ACIS (23). Limitations related to precision and risk of bias led to downgrading. The quality of evidence was considered **very low**.

Supplementary table 30: Communication and interaction skills

	Limitations	Assessment
**Consistency**	N/A	Not possible to assess as the data only came from one study
**Directness**	No serious limitations	Relevant study characteristics (population, intervention, comparison and outcome)
**Precision**	Very serious limitations	Data from one study, less than 800 participants
**Publication bias**	Not suspected	No findings of studies reported in trial registers not published
**Limitations in study design or execution**	Very serious limitations	High risk of bias

5.16. Extent of social support

Measured with a questionnaire (14). Limitations related to precision and risk of bias led to downgrading. The quality of evidence was considered **very low**.

Supplementary table 31: Extent of social support

	Limitations	Assessment
**Consistency**	N/A	Not possible to assess as the data only came from one study
**Directness**	No serious limitations	Relevant study characteristics (population, intervention, comparison and outcome)
**Precision**	Very serious limitations	Data from one study, less than 800 participants
**Publication bias**	Not suspected	No findings of studies reported in trial registers not published
**Limitations in study design or execution**	Very serious limitations	High risk of bias

5.17. Global cognitive function

Measured with MoCa (23). Limitations related to precision and risk of bias led to downgrading. The quality of evidence was considered **very low**.

Supplementary table 32: Global cognitive function

	Limitations	Assessment
**Consistency**	N/A	Not possible to assess as the data only came from one study
**Directness**	No serious limitations	Relevant study characteristics (population, intervention and outcome)
**Precision**	Very serious limitations	Data from one study, less than 800 participants
**Publication bias**	Not suspected	No findings of studies reported in trial registers not published
**Limitations in study design or execution**	Very serious limitations	High risk of bias

5.18. Lower body strength

Measured with CST (23). Limitations related to precision led to downgrading. The quality of evidence was considered **low**.

Supplementary table 33: Lower body strength

	Limitations	Assessment
**Consistency**	N/A	Not possible to assess as the data only came from one study
**Directness**	No serious limitations	Relevant study characteristics (population, intervention and outcome)
**Precision**	Very serious limitations	Data from one study, less than 800 participants
**Publication bias**	Not suspected	No findings of studies reported in trial registers not published
**Limitations in study design or execution**	No serious limitations	Low risk of bias

5.19. Agility

Measured with TUG (23). Limitations related to precision led to downgrading. The quality of evidence was considered **low**.

Supplementary table 34: Agility

	Limitations	Assessment
**Consistency**	N/A	Not possible to assess as the data only came from one study
**Directness**	No serious limitations	Relevant study characteristics (population, intervention and outcome)
**Precision**	Very serious limitations	Data from one study, less than 800 participants
**Publication bias**	Not suspected	No findings of studies reported in trial registers not published
**Limitations in study design or execution**	No serious limitations	Low risk of bias

5.20. Mobility

Measured with 5MWT (23). Limitations related to precision led to downgrading. The quality of evidence was considered **low**.

Supplementary table 35: Mobility

	Limitations	Assessment
**Consistency**	N/A	Not possible to assess as the data only came from one study
**Directness**	No serious limitations	Relevant study characteristics (population, intervention and outcome)
**Precision**	Very serious limitations	Data from one study, less than 800 participants
**Publication bias**	Not suspected	No findings of studies reported in trial registers not published
**Limitations in study design or execution**	No serious limitations	Low risk of bias

5.21. Quality of life

Measured with EQ-5D (7), SQLS and QLESQ (11), and with WHOQOL-BREEF (13, 25). Limitations related to consistency, precision and risk of bias led to downgrading. The quality of evidence was considered **very low.**

Supplementary table 36: Quality of life

	Limitations	Assessment
**Consistency**	Serious limitations	One study showed significant improvement for the intervention group compared with the control group for quality of life in general (25).One study showed significant improvement for the intervention group compared with the control group for quality of life related to utilization of leisure time. There was no significant difference between the groups for a range of other factors of quality of life. Significance within the groups was not stated (11). One study showed significant improvement, with Cohen's *d* -1.12 indicating large effect size, within the intervention group for quality of life related to social conditions. There was no significance within the control group, but the difference between the groups was not significant. Further, there were no significant findings in this study for quality of life related to other factors (13). One study showed significant improvement before Bonferroni correction within the intervention group, and not within the control group, for quality of life related to general health. No significance was found for the control group, but the difference between the groups was not significant. Furthermore, there were no significant findings for other domains of quality of life in this study (7).<br><br>It cannot be ruled out that heterogeneity regarding study characteristics can explain part of the inconsistency.
**Directness**	No serious limitations	Relevant study characteristics (population, intervention, comparison and outcome)
**Precision**	Serious limitations	Data from four studies, less than 800 participants
**Publication bias**	Not suspected	The material included both studies with significant findings and studies with non-significant findings. No findings of studies reported in trial registers not published.
**Limitations in study design or execution**	Very serious limitations	High risk of bias in two studies, serious risk of bias in one, some concern in one

5.22. Well-being

Measured with CHI (12). Limitations related to precision, directness and risk of bias led to downgrading. The quality of evidence was considered **very low.**

Supplementary table 37: Well-being

	Limitations	Assessment
**Consistency**	N/A	Not possible to assess as the data only came from one study
**Directness**	Serious limitations	Non-universal instrument
**Precision**	Very serious limitations	Data from one study, less than 800 participants
**Publication bias**	Not suspected	No findings of studies reported in trial registers not published
**Limitations in study design or execution**	Very serious limitations	High risk of bias

5.23. Patient experience (adherence)

Measured through calculation of proportion of attended sessions (7). Limitations related to directness, precision and risk of bias led to downgrading. The quality of evidence was considered **very low.**

Supplementary table 38: Patient experience (adherence)

	Limitations	Assessment
**Consistency**	N/A	Not possible to assess as the data only came from one study
**Directness**	Serious limitations	Non-adherence and adherence may be related to other factors than patient experience
**Precision**	Very serious limitations	Data from one study, less than 800 participants
**Publication bias**	Not suspected	No findings of studies reported in trial registers not published
**Limitations in study design or execution**	Very serious limitations	High risk of bias

6. Recommendations for further research

Supplementary table 39: Recommendations for further research

**Recommendation**	**Background/aim**
Higher number of participants	Detect potential effects on group level
Distinguish between results from participants with different diagnoses and interventions with different animal species	Strengthen of directness
State confidence interval	Provide more information about uncertainty of estimate (82)
State effect sizes	Further information regarding magnitude of effect is needed
Development of more standardized programs for intervention groups and control groups	Reduce confounding factors, provide more precise assessments of effectiveness
Development of intervention in tread with guidelines, for example from IAHAIO (6) for safety and welfare<br>As suggested in one of the studies (13):<br>Development of interventions addressing non-personal social behavior<br>Documentation of methods for overall safety, prevention of negative consequences and welfare for both participants and animals	Prevent potential negative consequences
Investigate physiological changes: Heart rate, blood pressure, HbA1c and lipid levels	It is stated that patients with schizophrenia are at high risk for development of metabolic syndrome (83). Significant lower levels of cortisol were found within the intervention group in one of the included studies (7). Effect related to cardiovascular risk factors has been found for a varied population, but there is a need for further research (84)
Investigate effects related to motivation in general, for participation in intervention and for physical activity:<br>State reasons for non-adherence<br>State reasons for lost to follow-up<br>Use of validated instruments, such as IMI-SR (85)	Apathy is one of the negative symptoms associated with schizophrenia (86)
Investigate effectiveness on cognitive function through validated instruments, such as TMT B (87)	Only one of the studies directly assessed cognitive function (23). This will be highly relevant to investigate as it is a core feature of schizophrenia and an important determinant of disability (88, 89)
Examination of non-verbal communication through validated instruments, such as BGRS (9)	Non-verbal communication, measured with BGRS, was examined in one of the studies. The study showed improvement, but significance was not investigated due to low number of participants (9)
Investigate differences in effect across symptom groups using categorization of effect size, such as SRD<br>Investigate effect for specific symptoms, such as anhedonia<br>Comparison of treatment effect in different contexts, for example inpatient settings compared with day-care settings<br>Divide patients in treatment groups based on similar symptoms or other characteristics, and compare differences in effect on group level	Development of more personalized treatment

Abbreviations:
BGRS = The Budapest Gesture Rating Scale
HbA1c = Hemoglobin A1c
IMI-SR = The Intrinsic Motivation Inventory for Schizophrenia Research
SRD = Sum of ranking differences
TMT B = Trail making test part B

8. References

1. Page MJ, McKenzie JE, Bossuyt PM, Boutron I, Hoffmann TC, Mulrow CD, et al. The PRISMA 2020 statement: an updated guideline for reporting systematic reviews. BMJ. 2021;372:n71.
2. Lefebvre C, Glanville J, Briscoe S, Littlewood A, Marshall C, Metzendorf M-I, et al. Chapter 4: Searching for and selecting studies In: Higgins JPT, Thomas J, Chandler J, Cumpston M, Li T, Page MJ, Welch VA (editors). Cochrane Handbook for Systematic Reviews of Interventions 2021 [cited 2021 30.09]. Available from: https://training.cochrane.org/handbook/current/chapter-04.
3. Område for helsetjenester i Folkehelseinstituttet. Slik oppsummerer vi forskning. Håndbok for Folkehelseinstituttet. 2018 [cited 2021 30.09]. Available from:

https://www.fhi.no/globalassets/dokumenterfiler/rapporter/2018/slik-oppsummerer-vi-forskning-2018v2-endret-2021.pdf.
4. Hawkins EL, Lawrie SM, Hawkins RD, Dennis M, Williams JM. Animal-assisted therapy for schizophrenia and related disorders: A systematic review. Journal of Psychiatric Research. 2019;115:51-60.
5. O'Haire M, Guérin N, Kirkham A. Animal-Assisted Intervention for trauma: a systematic literature review. Frontiers in Psychology. 2015;6:1121.
6. IAHAIO. White Paper on Animal-Assisted Interventions; IAHAIOS definisjoner for dyreassisterte intervensjoner og retningslinjer for å ivareta dyrenes velferd: IAHAIO; 2014 [updated 2018; cited 2021 23.09]. Available from: https://iahaio.org/wp/wp-content/uploads/2021/01/iahaio-white-paper-2020rev2018-norwegian.pdf.
7. Calvo P, Fortuny JR, Guzman S, Macias C, Bowen J, Garcia ML, et al. Animal Assisted Therapy (AAT) Program As a Useful Adjunct to Conventional Psychosocial Rehabilitation for Patients with Schizophrenia: Results of a Small-scale Randomized Controlled Trial. FRONTIERS IN PSYCHOLOGY. 2016;7.
8. Kovacs Z, Kis R, Rozsa S, Rozsa L. Animal-assisted therapy for middle-aged schizophrenic patients living in a social institution. A pilot study. Clinical Rehabilitation. 2004;18(5):483-6.
9. Kovacs Z, Bulucz J, Kis R, Simon L. An exploratory study of the effect of animal-assisted therapy on nonverbal communication in three schizophrenic patients. ANTHROZOOS. 2006;19(4):353-64.
10. Lang UE, Jansen JB, Wertenauer F, Gallinat J, Rapp MA. Reduced anxiety during dog assisted interviews in acute schizophrenic patients. European journal of integrative medicine. 2010;2(3):123-7.
11. Nathans-Barel I, Feldman P, Berger B, Modai I, Silver H. Animal-Assisted Therapy Ameliorates Anhedonia in Schizophrenia Patients. Psychotherapy and Psychosomatics. 2005;74(1):31-5.
12. Chen T-T, Hsieh T-L, Chen M-L, Tseng W-T, Hung C-F, Chen C-R. Animal-Assisted Therapy in Middle-Aged and Older Patients With Schizophrenia: A Randomized Controlled Trial. Frontiers in psychiatry. 2021;12:713623.
13. Villalta-Gil V, Roca M, Gonzalez N, Domenec E, Cuca, Escanilla A, et al. Dog-assisted therapy in the treatment of chronic schizophrenia inpatients. Anthrozoos. 2009;22(2):149-59.
14. Chu C, Liu C, Sun C, Lin J. The effect of animal-assisted activity on inpatients with schizophrenia. Journal of Psychosocial Nursing & Mental Health Services. 2009;47(12):42-8.
15. Universitetsbiblioteket. Medisin og helsefag n.d. [cited 2021 30.09]. Available from: https://bibsys-almaprimo.hosted.exlibrisgroup.com/primo-explore/dbsearch?query=contains,dbcategory,&tab=jsearch_slot&sortby=title&vid=UBB&lang=no_NO&offset=0&databases=category,Medical%20sciences.
16. Universitetsbiblioteket. Psykologi og pedagogikk n.d. [cited 2021 30.09]. Available from: https://bibsys-almaprimo.hosted.exlibrisgroup.com/primo-explore/dbsearch?query=contains,dbcategory,&tab=jsearch_slot&sortby=title&vid=UBB&lang=no_NO&offset=0&databases=category,Psychology%20and%20pedagogy.
17. Karolinska Institutet University Library. Systematic reviews n.d. [cited 2021 30.09]. Available from: https://kib.ki.se/en/search-evaluate/systematic-reviews.
18. Affinity foundation. The Chair: research Spain n.d. [cited 2022 18.06]. Available from: https://www.fundacion-affinity.org/en/foundation/research#publicaciones.
19. IAHAIO. Articles published n.d. [cited 2022 18.06]. Available from: https://iahaio.org/journal/articles-published/.
20. Therapy animals vzw. Reading material 2019 [cited 2022 25.06]. Available from: https://therapiedier.be/en/reading-material/.
21. McGowan J, Sampson M, Salzwedel DM, Cogo E, Foerster V, Lefebvre C. PRESS Peer Review of Electronic Search Strategies: 2015 Guideline Statement. Journal of Clinical Epidemiology. 2016;75:40-6.

22. Barker SB, Dawson KS. The effects of animal-assisted therapy on anxiety ratings of hospitalized psychiatric patients. Psychiatric services (washington, DC). 1998;49(6):797-801.
23. Chen CR, Hung CF, Lee YW, Tseng WT, Chen ML, Chen TT. Functional Outcomes in a Randomized Controlled Trial of Animal-Assisted Therapy on Middle-Aged and Older Adults with Schizophrenia. Int J Environ Res Public Health. 2022;19(10).
24. Monfort M, Benito A, Haro G, Fuertes-Saiz A, Cañabate M, Baquero A. The Efficacy of Animal-Assisted Therapy in Patients with Dual Diagnosis: Schizophrenia and Addiction. Int J Environ Res Public Health. 2022;19(11).
25. Shih C-A, Yang M-H. Effect of animal-assisted therapy (AAT) on social interaction and quality of life in patients with schizophrenia during the COVID-19 pandemic: An experimental study. Asian Nurs Res (Korean Soc Nurs Sci). 2023.
26. Barker SB, Wolen AR. The Benefits of Human-Companion Animal Interaction: A Review. JOURNAL OF VETERINARY MEDICAL EDUCATION. 2008;35(4):487-95.
27. Bernabei V, De Ronchi D, La Ferla T, Moretti F, Tonelli L, Ferrari B, et al. Animal-assisted interventions for elderly patients affected by dementia or psychiatric disorders: A review. Journal of Psychiatric Research. 2013;47(6):762-73.
28. Cakici A, Kok M. Animal assisted therapy. Psikiyatride Guncel Yaklasimlar. 2020;12(1):117-30.
29. Charry-Sanchez JD, Pradilla I, Talero-Gutierrez C. Animal-assisted therapy in adults: A systematic review. Complementary therapies in clinical practice. 2018;32:169-80.
30. Cirulli F, Borgi M, Berry A, Francia N, Alleva E. Animal-assisted interventions as innovative tools for mental health. ANNALI DELL ISTITUTO SUPERIORE DI SANITA. 2011;47(4):341-8.
31. Cherniack EP, Cherniack AR. The benefit of pets and animal-assisted therapy to the health of older individuals. Current gerontology and geriatrics research. 2014;2014:623203.
32. Dimitrijevic I. Animal-assisted therapy - A new trend in the treatment of children and addults. Psychiatria Danubina. 2009;21(2):236-41.
33. Hawkins EL, Hawkins RD, Dennis M, Williams JM, Lawrie SM. Animal-assisted therapy for schizophrenia and related disorders: A systematic review. Journal of psychiatric research. 2019;115:51-60.
34. Kamioka H, Okada S, Tsutani K, Park H, Okuizumi H, Handa S, et al. Effectiveness of animal-assisted therapy: A systematic review of randomized controlled trials. Complementary therapies in medicine. 2014;22(2):371-90.
35. Koukourikos K, Georgopoulou A, Kourkouta L, Tsaloglidou A. Benefits of Animal Assisted Therapy in Mental Health. International Journal of Caring Sciences. 2019;12(3):1898-905.
36. Lasa SM, Ferriero G, Brigatti E, Valero R, Franchignoni F. Animal-assisted interventions in internal and rehabilitation medicine: a review of the recent literature. PANMINERVA MEDICA. 2011;53(2):129-36.
37. Maujean A, Pepping CA, Kendall E. A systematic review of randomized controlled trials of animal-assisted therapy on psychosocial outcomes. Anthrozoos. 2015;28(1):23-36.
38. Peluso S, De Rosa A, De Lucia N, Antenora A, Illario M, Esposito M, et al. Animal-Assisted Therapy in Elderly Patients: Evidence and Controversies in Dementia and Psychiatric Disorders and Future Perspectives in Other Neurological Diseases. Journal of geriatric psychiatry and neurology. 2018;31(3):149-57.
39. Rossetti J, King C. Use of Animal-Assisted Therapy with Psychiatric Patients - A Literature Review. JOURNAL OF PSYCHOSOCIAL NURSING AND MENTAL HEALTH SERVICES. 2010;48(11):44-8.
40. Spattini L, Mattei G, Raisi F, Ferrari S, Pingani L, Galeazzi GM. Efficacy of animal assisted therapy on people with mental disorders: An update on the evidence. Minerva Psichiatrica. 2018;59(1):54-66.
41. Virues-Ortega J, Pastor-Barriuso R, Castellote JM, Poblacion A, de Pedro-Cuesta J. Effect of animal-assisted therapy on the psychological and functional status of elderly populations and patients with psychiatric disorders: A meta-analysis. Health Psychology Review. 2012;6(2):197-221.

42. Barak Y, Savor O, Mavashev S, Beni A. Animal assisted therapy for elderly schizophrenic patients: A one year controlled trial. ISRAEL JOURNAL OF PSYCHIATRY AND RELATED SCIENCES. 2001;38(1):69-.
43. Barker SB, Pandurangi AK, Best AM. Effects of Animal-Assisted Therapy on Patients' Anxiety, Fear, and Depression Before ECT. The Journal of ECT. 2003;19(1).
44. Barnes DJ. The effects of pet facilitated therapy on social self care behaviors of schizophrenics. Dissertation Abstracts International Section A: Humanities and Social Sciences. 1986;47(2-A):419.
45. Bauman L, Posner, M., Sachs, K., & Szita, R. . The effects of animal-assisted therapy on communication patterns with chronic schizophrenics. The Latham Letter, 13(4) 3-5. 1991.
46. Brown S, Snelders J, Godbold J, Moran-Peters J, Driscoll D, Donoghue D, et al. Effects of Animal-Assisted Activity on Mood States and Feelings in a Psychiatric Setting. Journal of the American Psychiatric Nurses Association. 2019;26(6):555-67.
47. Chang BH, Chang SY, Yeh ML. The healing process of chronic schizophrenic patients in animal-assisted therapy. Chin Group Psychother. 2015 21(3): 47-60.
48. Corson SA, Corson EL, Gwynne PH, Arnold LE. Pet dogs as nonverbal communication links in hospital psychiatry. Compr Psychiatry. 1977;18(1):61-72.
49. Sterne JAC, Hernán MA, Reeves BC, Savović J, Berkman ND, Viswanathan M, et al. ROBINS-I: a tool for assessing risk of bias in non-randomised studies of interventions. BMJ. 2016;355:i4919.
50. Cox C. Pet-facilitated occupational therapy: Efficacy in a psychiatric setting [M.S.]. Ann Arbor: San Jose State University; 1999.
51. Eklund A, Johansson A. "Det här är Hilda, hon är Uppsalas bästa tjej"- En fallstudie om vårdhundens roll i socialt arbete [Student thesis]2014.
52. Hall P, Malpus Z. Pets as therapy: Effects on social interaction in long-stay psychiatry. British journal of nursing (Mark Allen Publishing). 2000;9:2220-5.
53. Haughie E, Milne D, Elliott V. An evaluation of companion pets with elderly psychiatric patients. Behavioural Psychotherapy. 1992;20(4):367-72.
54. Henry CL, Crowley SL. The Psychological and Physiological Effects of Using a Therapy Dog in Mindfulness Training. Anthrozoös. 2015;28(3):385-402.
55. Holcomb R, Meacham M. Effectiveness of an Animal-Assisted Therapy Program in an Inpatient Psychiatric Unit. Anthrozoös. 1989;2(4):259-64.
56. Hudson NJ. Animal assisted therapy and the effects on anxiety and behavioral symptoms for geriatric patients living in a facility [D.N.P.]. Ann Arbor: Northern Kentucky University; 2016.
57. Kung SM, Lan TH, Chen WC, Lin SC, Tseng ML, Chiu HJ. The evaluation of pet therapy on negative symptoms in inpatients with chronic schizophrenia. 158th annual meeting of the american psychiatric association; 2005 may 21-26; atlanta, georgia, USA. 2005.
58. LaJoie K. An evaluation of the effectiveness of using animals in therapy. Thesis dissertation. Psy D Spalding University. 2003.
59. Levinson BM. The dog as a "co-therapist.". Mental Hygiene New York. 1962;46:59-65.
60. Marques MI, Mendes AMdOC, Gamito ARF, Sousa L, editors. Effectiveness of animal-assisted interventions in preventing violence in acute psychiatric inpatients2015.
61. Marr CA, French L, Thompson D, Drum L, Greening G, Mormon J, et al. Animal-Assisted Therapy in Psychiatric Rehabilitation. Anthrozoös. 2000;13(1):43-7.
62. Montolio MM, Sancho-Pelluz J. Animal-assisted therapy in the residential treatment of dual pathology. International Journal of Environmental Research and Public Health. 2020;17(1):120.
63. Moretti F, Bernabei V, Bonafede R, Forlani C, De Ronchi D, Atti AR, et al. A pet therapy intervention on elderly inpatients: An epidemiological study. European Psychiatry. 2010;25(SUPPL. 1).
64. Moretti F, De Ronchi D, Bernabei V, Ferrari B, Forlani C, Atti AR, et al. Pet therapy in elderly patients with mental illness. Psychogeriatrics. 2011;11(2):125-9.

65. Morgan TD. An examination of the anxiolytic effects of interaction with a therapy dog [Psy.D.]. Ann Arbor: Indiana University of Pennsylvania; 2008.
66. Mulvaney-Roth P, Jackson C, Bert L, Eriksen S, Ryan M. Using Pet Therapy to Decrease Patients' Anxiety on Two Diverse Inpatient Units. Journal of the American Psychiatric Nurses Association. 2022:1078390321999719.
67. Nagendrappa S, Shoib S, Rehman S, Grigo O, Ransing R. Recognizing the role of animal-assisted therapies in addressing mental health needs during the COVID-19 pandemic. Asian Journal of Psychiatry. 2020;53:102390.
68. Nepps P, Stewart CN, Bruckno SR. Animal-Assisted Activity: Effects of a Complementary Intervention Program on Psychological and Physiological Variables. J Evid Based Complementary Altern Med. 2014;19(3):211-5.
69. Nurenberg JR, Schleifer SJ, Shaffer TM, Yellin M, Desai PJ, Amin R, et al. Animal-assisted therapy with chronic psychiatric inpatients: equine-assisted psychotherapy and aggressive behavior. Psychiatric services (Washington, DC). 2015;66(1):80-6.
70. Pollock M, Williams RE, Gomez SM. Animals as icebreakers: A pilot animal-assisted therapy group for veterans with serious mental illness. International Journal of Psychosocial Rehabilitation. 2017;21:123-35.
71. Saludes C. Canines in Clinical Settings: Assessing the Psychosocial Outcomes of Therapy Dog Inclusion during a Stressful Interview [M.S.Psy.]. Ann Arbor: Azusa Pacific University; 2018.
72. Stefanini MC, Martino A, Allori P, Galeotti F, Tani F. The use of Animal-Assisted Therapy in adolescents with acute mental disorders: A randomized controlled study. Complementary Therapies in Clinical Practice. 2015;21(1):42-6.
73. Thompson M, Kennedy RW, Igou S. Pets as socializing agents with chronic psychiatric patients: An initial study. . In: Katcher, Aaron H/Beck, Alan M (Hrsg): New Perspectives on Our Lives with Companion Animals Philadelphia: University of Pennsylvania 1983, 427-430. 1983.
74. Tribulato J. Animal -assisted therapy for uncommunicative psychiatric patients with catatonic features [Psy.D.]. Ann Arbor: Pepperdine University; 2004.
75. Venkatesan P. Pawsonalised medicine: animals for mental health. The Lancet Psychiatry. 2019;6(3):205-6.
76. Villalta-Gil V, Ochoa S, Vilaplana M, Haro JM, Usall J. Pet-assisted therapy for inpatients with diagnosis of schizophrenia. ACTA PSYCHIATRICA SCANDINAVICA. 2006;114:49-.
77. Zisselman MH, Rovner BW, Shmuely Y, Ferrie P. A pet therapy intervention with geriatric psychiatry inpatients. Am J Occup Ther. 1996;50(1):47-51.
78. Hoffmann TC, Glasziou PP, Boutron I, Milne R, Perera R, Moher D, et al. Better reporting of interventions: template for intervention description and replication (TIDieR) checklist and guide. BMJ : British Medical Journal. 2014;348:g1687.
79. Schünemann H, Brożek J, Guyatt G, Oxman A. GRADE handbook for grading quality of evidence and strength of recommendations, 5. Quality of evidence: The GRADE Working Group 2013 [cited 2021 30.09]. Available from: https://gdt.gradepro.org/app/handbook/handbook.html.
80. Guyatt G, Oxman AD, Kunz R, Brozek J, Alonso-Coello P, Rind D, et al. Corrigendum to GRADE guidelines 6. Rating the quality of evidence-imprecision. J Clin Epidemiol 2011;64:1283–1293. Journal of Clinical Epidemiology. 2021;137:265.
81. Murad MH, Mustafa RA, Schünemann HJ, Sultan S, Santesso N. Rating the certainty in evidence in the absence of a single estimate of effect. Evidence Based Medicine. 2017;22(3):85.
82. Schünemann H, Vist G, Higgins J, Santesso N, Deeks J, Glasziou P, et al. Chapter 15: Interpreting results and drawing conclusions. In: Higgins JPT, Thomas J, Chandler J, Cumpston M, Li T, Page MJ, Welch VA (editors). Cochrane Handbook for Systematic Reviews of Interventions version 6.22021 [updated 02.21; cited 2021 30.09]. Available from: www.training.cochrane.org/handbook
83. Mitchell AJ, Vancampfort D, Sweers K, van Winkel R, Yu W, De Hert M. Prevalence of metabolic syndrome and metabolic abnormalities in schizophrenia and related disorders--a systematic review and meta-analysis. Schizophrenia bulletin. 2013;39(2):306-18.

84. Rathish D, Rajapakse RPVJ, Weerakoon KGAD. The role of cortisol in the association of canine-companionship with blood pressure, glucose, and lipids: a systematic review. High Blood Pressure & Cardiovascular Prevention. 2021;28(5):447-55.
85. Choi J, Mogami T, Medalia A. Intrinsic Motivation Inventory: An Adapted Measure for Schizophrenia Research. Schizophrenia Bulletin. 2010;36(5):966-76.
86. Helsedirektoratet. Utredning, behandling og oppfølging av personer med psykoselidelser 2013 [cited 2021 23.09]. Available from: https://www.helsedirektoratet.no/retningslinjer/psykoselidelser/Utredning,%20behandling%20og%20oppf%C3%B8lging%20av%20personer%20med%20psykoselidelser%20%E2%80%93%20Nasjonal%20faglig%20retningslinje%20(fullversjon).pdf/_/attachment/inline/a2c5a070-19d8-47df-b86c-9e9e6002c514:643b749f68005e7572f8e70b242c0f0af6f17910/Utredning,%20behandling%20og%20oppf%C3%B8lging%20av%20personer%20med%20psykoselidelser%20%E2%80%93%20Nasjonal%20faglig%20retningslinje%20(fullversjon).pdf.
87. Laere E, Tee SF, Tang PY. Assessment of Cognition in Schizophrenia Using Trail Making Test: A Meta-Analysis. Psychiatry Investig. 2018;15(10):945-55.
88. Harvey PD, Strassnig MT, Silberstein J. Prediction of disability in schizophrenia: Symptoms, cognition, and self-assessment. Journal of Experimental Psychopathology. 2019;10(3):2043808719865693.
89. McCutcheon RA, Reis Marques T, Howes OD. Schizophrenia—An Overview. JAMA Psychiatry. 2020;77(2):201-10.
